# The Development of a Checklist to Enhance Methodological Quality in Intervention Programs

**DOI:** 10.3389/fpsyg.2016.01811

**Published:** 2016-11-18

**Authors:** Salvador Chacón-Moscoso, Susana Sanduvete-Chaves, Milagrosa Sánchez-Martín

**Affiliations:** ^1^HUM-649 Innovaciones Metodológicas en Evaluación de Programas, Departamento de Psicología Experimental, Facultad de Psicología, Universidad de SevillaSevilla, Spain; ^2^Universidad Autónoma de ChileSantiago de Chile, Chile; ^3^Department of Psychology, Universidad Loyola AndaluciaSevilla, Spain

**Keywords:** checklist, methodological quality, content validity, inter-coder reliability, primary studies

## Abstract

The methodological quality of primary studies is an important issue when performing meta-analyses or systematic reviews. Nevertheless, there are no clear criteria for how methodological quality should be analyzed. Controversies emerge when considering the various theoretical and empirical definitions, especially in relation to three interrelated problems: the lack of representativeness, utility, and feasibility. In this article, we (a) systematize and summarize the available literature about methodological quality in primary studies; (b) propose a specific, parsimonious, 12-items checklist to empirically define the methodological quality of primary studies based on a content validity study; and (c) present an inter-coder reliability study for the resulting 12-items. This paper provides a precise and rigorous description of the development of this checklist, highlighting the clearly specified criteria for the inclusion of items and a substantial inter-coder agreement in the different items. Rather than simply proposing another checklist, however, it then argues that the list constitutes an assessment tool with respect to the representativeness, utility, and feasibility of the most frequent methodological quality items in the literature, one that provides practitioners and researchers with clear criteria for choosing items that may be adequate to their needs. We propose individual methodological features as indicators of quality, arguing that these need to be taken into account when designing, implementing, or evaluating an intervention program. This enhances methodological quality of intervention programs and fosters the cumulative knowledge based on meta-analyses of these interventions. Future development of the checklist is discussed.

## Introduction

Meta-analyses and systematic reviews aim to summarize the literature and generalize the results from a series of different studies about a given area of interest (Cheung, 2015). To avoid biased or erroneous conclusions, this requires clear criteria regarding the methodological quality of the primary studies and how to combine or analyze studies of different methodological quality (Jüni et al., 2001). Although, there is a general consensus about this need (Moher et al., 1996; Altman et al., 2001), a number of controversies arise when studying methodological quality in practice. For example, is it possible to give a one-dimensional answer to what is probably a multidimensional problem? Do we have clear criteria for deciding which specific and differently weighted methodological quality items should be considered? Which criteria should be used to decide between methodological quality indexes based on scores obtained from just one item or from a global assessment of several weighted items? Is it worthwhile trying to study a general construct that might not be equally applicable to all the contexts in which it might be used?

Despite this complexity, the extensive literature on these issues is testament to the importance of considering the methodological quality of primary studies. The present paper reviews the work in this area until July 2015. We begin by summarizing the relevant literature and then introduce the main problems derived from the state of the art.

### Theoretical and Empirical Definition of *Methodological Quality*

The concept of *methodological quality* is complex and multidimensional. It has been defined theoretically from different perspectives, such as (a) internal validity (Moher et al., 1996); (b) external validity (Rubinstein et al., 2007); (c) both internal and external validity (Jüni et al., 2001); (d) internal, external, statistical, and construct validity (Valentine and Cooper, 2008); (e) precision of the study report (Moher et al., 1998; Altman et al., 2001; Efficace et al., 2006; Hopewell et al., 2006; Rutjes et al., 2006; Cornelius et al., 2009; Li et al., 2009); (f) appropriate statistical analysis (Minelli et al., 2007); (g) ethical implications (Jüni et al., 1999); (h) relevance for the intervention area (Sargeant et al., 2006; Jefferson et al., 2009; Jiménez-Requena et al., 2009); or (i) publication status (Moher et al., 2009).

This theoretical diversity of the concept of *methodological quality* leads to different approaches to measuring it empirically. The main approaches described in the literature are:

• Scales. These can be defined as validated tools used to measure the construct. At least the content, construct, and criterion validity evidence should be tested (Crocker and Algina, 1986; American Educational Research Association, American Psychological Association, and National Council on Measurement in Education, 1999; Abad et al., 2011). They are usually structured into different dimensions comprising differently weighted items (Sanderson et al., 2007). These items are either summed to obtain a global index (Jadad et al., 1996; Classen et al., 2008) or yield various indexes based on the dimensions considered (Jefferson et al., 2009).
• Checklists. The main difference between these tools and scales is that checklists have not been tested through an extensive validation process. Partial validity evidence may be presented, for example, based only on content or construct validity evidence. Checklists may also propose a final global index (Effective Public Health Practice Project, 1998; Efficace et al., 2003; Sanderson et al., 2007; Pluye et al., 2009); just one individual component (Gilbody et al., 2007); or several components (Bossuyt et al., 2003; Taji et al., 2006; Schulz et al., 2010).
• General recommendations. These take the form of advice, including general aspects to consider when assessing methodological quality. They may sometimes describe just a few examples of possible items, without specifying a whole list of proposed items. In sum, recommendations refer to those approaches that do not fulfill the criteria required by the previous two categories (Ford and Moayyedi, 2009; Linde, 2009; Wilson, 2009).

At this point, it is interesting to mention the difference between *quality in primary studies* and *quality of the report of primary studies* (Leonardi, 2006). It is very important to study the quality of the report of primary studies because the study of quality in primary studies is mostly based on reports given by authors. Indeed, this is usually the only source to obtain information about primary studies (Altman et al., 2001; Grimshaw et al., 2006; Cornelius et al., 2009). Nevertheless, we base our study on quality of primary studies (instead of the report) to (a) give researchers guidelines to check the methodological quality of studies included in a meta-analysis, to facilitate conclusions about possible risk of bias in the conclusions; (b) provide practitioners with a checklist to enhance methodological quality when designing, implementing, and evaluating their interventions; and (c) make explicit the criteria for why we included some concrete items and excluded others from an available extensive list. This information can be useful in case researchers or practitioners are interested in including different items from the extensive list based on their aims and specific contexts.

### Problems Derived from the Dispersion in the Definition of *Methodological Quality*

The abovementioned characteristics of the concept of *methodological quality*, that is, the diversity in its theoretical and empirical definition (Linde, 2009), imply three interrelated and specific problems:

**Lack of *representativeness*** (R), the extent to which the specific item represents the methodological quality domain to which it is assigned. There are no clear criteria for choosing the optimal tool to measure methodological quality. This occurs especially since it is common to use non-randomized studies in social sciences (Shadish et al., 2005). This is due to a shortage of instruments that (a) are rigorously developed and (b) have reliability and/or validity evidence with tested R (Crowe and Sheppard, 2011). Their use is based on criteria that have no empirical support (Valentine and Cooper, 2008). For example, some authors opt to use individual components (Field et al., 2014; Eken, 2015). Other authors apply scales that provide a global value, even when they are strongly criticized for the lack of a bias estimation (Crowe and Sheppard, 2011). In spite of this, many scales are available and used nowadays (Dechartres et al., 2011). As a consequence, different scales applied to the same group of studies may indicate different levels of methodological quality (Greenland and O’Rourke, 2001; Jüni et al., 2001). Furthermore, some tools might be labeled as scales but without providing information about their construction process (Taji et al., 2006; Jefferson et al., 2009).

**Lack of *utility*** (U), the extent to which the specific item is useful for assessing the methodological quality of the study with respect to the assigned domain. In practice, scales usually include many items susceptible to omission because they are not relevant or essential for measuring the construct. Therefore, they could be shortened (Jüni et al., 2001; Conn and Rantz, 2003).

**Lack of *feasibility*** (F), the extent to which data codification is viable because data are available and can be gathered. Tools to measure methodological quality are usually complex and their items lack operational specificity. As a consequence, they are hard to understand and require previous training for coders. Additionally, the information needed is in most cases unavailable (Classen et al., 2008; Valentine and Cooper, 2008).

### Objectives

To resolve the aforementioned problems when measuring methodological quality, the objectives of this paper are (a) to systematize and summarize the available literature about methodological quality in primary studies published until July 2015 (Study 1: systematic review); (b) to propose a specific, parsimonious checklist to empirically define the methodological quality of primary studies in meta-analyses and systematic reviews. This tool offers evidence of good R, U, and F based on expert judges (Study 2: content validity); and (c) to present evidence of adequate inter-coder reliability in the items that form the checklist (Study 3).

### Contributions of this Study Compared to Other Studies Available in the Literature

The most popular tools to measure methodological quality present some of these problems. For example, the study Design and Implementation Assessment Device (DIAD) (Valentine and Cooper, 2008) was systematically developed. Nevertheless, it did not present reliability and validity evidence (weak R), and its application was complex (weak F).

Another example is the Cochrane Collaboration’s tool for assessing risk of bias in randomized trials. It focuses on individual biases (Higgins et al., 2011). In this case, we did not find reliability and validity evidence (weak R). Furthermore, there was lack of U in social sciences because it is only applicable for randomized control trials (Shadish et al., 2005). Finally, at least two of the items (incomplete outcome data and selective reporting) are difficult to assess (weak F).

The Physiotherapy Evidence Database quality scale for randomized control trials —the PEDro scale— (Sherrington et al., 2000) presents reliability (Maher et al., 2003) and validity (Macedo et al., 2010) evidence (good in R). A website^1^ offers access to the tool and a training program for raters (good in F). Nevertheless, it lacks U for our proposal because it is an adequate tool only for randomized control trials and only in the context of physiotherapy.

The checklist for the assessment of methodological quality presented by Downs and Black (1998) is good in U because it can be applied to randomized and non-randomized studies. Nevertheless, it partially presents weaknesses in R because, although it presents validity evidence, it attains poor reliability in a subscale and some specific items. Furthermore, practitioners who are not experts in methodology might experience some problems in its application (weak F).

The Newcastle–Ottawa Scale (NOS) for assessing the quality of non-randomized studies in a meta-analysis (Wells et al., 2009) presents good F: the tool and its manual are freely accessible through the Internet. Nevertheless, its R is medium because it presents intra-rater reliability and content and criterion validity but its construct validity has not been established yet. In addition, its U can be considered medium because it has been tested exclusively to be applied to non-randomized studies, but we do not know how it works for randomized studies.

There are quite well-developed tools that measure the quality of the report of primary studies, indicating the aspects to be made explicit when reporting a study, but without valuing the actions to improve the methodological quality of a study or intervention. Some of them are (Portell et al., 2015) (a) the Consolidated Standards of Reporting Trials (CONSORT) statement (Schulz et al., 2010) for randomized control trials; (b) the STrengthening the Reporting of OBservational Studies in Epidemiology (STROBE) statement (von Elm et al., 2007); (c) Guidelines for Reporting Momentary Studies (Stone and Shiffman, 2002) for intensive repeated measurements in naturalistic settings; (d) Guidelines for Qualitative Research Methodologies (Blignault and Ritchie, 2009); (e) Guidelines for Conducting and Reporting Mixed Research for Counselor Researchers (Leech and Onwuegbuzie, 2010); and (f) Guidelines for Reporting Evaluations Based on Observational Methodology (Portell et al., 2015) for low intervention designs. Our proposal is to measure the methodological quality of primary studies instead of the report of these studies. Consequently, our aim and the aim of the previously mentioned tools are clearly different. They both can be considered complementary because the methodological quality of a study cannot be valued when the aspects to evaluate are not reported.

Literature reviews about methodological quality have already been done (e.g., Donegan et al., 2010). Furthermore, tools to measure methodological quality with good results in inter-rater reliability and content validity already exist (e.g., Wells et al., 2009). This paper integrates both contributions: it updates the literature reviews until July 2015 exhaustively providing a list of the most frequent quality items; and based on the results, proposes a tool to enhance methodological quality with content validity (R, U, and F of items) and inter-rater reliability evidence.

In sum, our proposed 12-items checklist addresses the limitations that the other proposals present in total or partially. First, it presents R, U, and F evidence for each of its items based on a systematic literature review and content validity study. Second, appropriate results in reliability can be considered an additional evidence of R and F. In that case, we can describe our items as operationally specified, easy to be applied, and understandable. Third, additional U evidence of the tool is its applicability in different designs (randomized and non-randomized) and different contexts (it can be applied in the design, intervention, and/or evaluation of any program). Forth, additional F evidence is the transparency in procedure and results (presented objectively, thoughtfully, and in detail). We made explicit (a) the inclusion and exclusion criteria applied in each stage of the development of the tool; (b) the papers, tools, and items found in the literature; (c) the values obtained in the content validity study in R, U, and F for the most frequently used items to measure quality; and (d) the reliability coefficients. Finally, the proposed tool measures methodological quality instead of the quality of the report in methodological aspects.

## Study 1. Systematic Review to Search for Methodological Quality Indicators

### Method

#### Inclusion and Exclusion Criteria

We searched for papers published up to July 2015. Four inclusion criteria were applied: (a) methodological quality in primary studies was measured, (b) the full text was available, (c) it was written in English or Spanish, and (d) the instrument used to measure methodological quality was not previously included (was original, not repeated).

#### Information to Code

Tools to measure methodological quality in primary studies were identified. After that, they were assigned to the previously defined categories regarding the empirical definition of methodological quality: scales, checklists, and general recommendations.

Subsequently, the most frequently used items in the previously identified tools were compiled by two independent researchers. This item gathering was exhaustive but not necessarily mutually exclusive; that is, different items could refer to the same methodological quality content but define it with different degrees of detail/accuracy. Any redundancies in this regard would be removed in the content validity study (Study 2).

Finally, items were assigned to different dimensions and sub-dimensions based on a categorization of moderator variables in meta-analyses (Lipsey, 1994; Sánchez-Meca, 1997; Sánchez-Meca et al., 1998; Merrett et al., 2013): (a) substantive characteristics, pertinent to characterizing the phenomenon under study and referring to three aspects: subject characteristics (description of participants such as gender, age, or cultural status), the setting in which the intervention was implemented (e.g., geographical, cultural, temporal, or political context), and the nature of the intervention provided (e.g., modality, underlying theory, duration or number of sessions); (b) methodological or procedural aspects, referring to the manner in which the study was conducted (i.e., variations in the design, research procedures, quality of measures, and forms of data analysis); and (c) characteristics extrinsic to both the substantive phenomenon and the research methods. This includes characteristics of the researcher(s) (e.g., gender or affiliation), research circumstances (e.g., sponsorship), or reporting (e.g., form of publication or accuracy of the reporting). It has been reported that these variables are correlated with the magnitude of the effect in many meta-analyses (Lipsey, 1994).

#### Search Strategies

The search was carried out in 12 databases that were of interest due to their content. Specifically, these were Web of Science, Scopus, Springer, EBSCO Online, Medline, CINAHL, Econlit, MathSci Net, Current Contents, Humanities Index, ERIC, and PsycINFO.

The keywords were “methodological quality” AND “meta-analysis” AND “primary studies.” Title, abstract, keywords, and full text were examined. In addition, the reference lists of studies found were checked to identify other studies of interest. This procedure was repeated until no further relevant studies were discovered.

#### Coding Procedures

Inter-coder reliability (Nimon et al., 2012; Stolarova et al., 2014) was studied. The degree of agreement between two researchers (two of the authors, CM and SC) was calculated using Cohen’s κ coefficient. Any disagreements were resolved by consensus.

### Results

**FIGURE 1:**
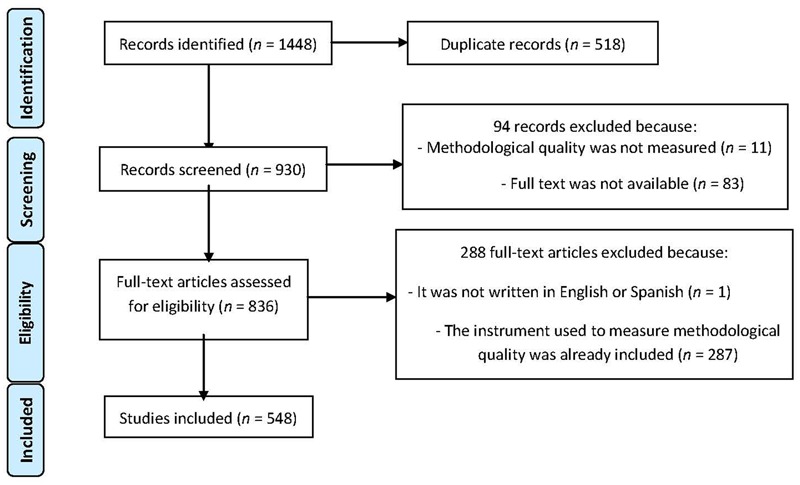
**Flow chart in the search for papers (Moher et al., 2009)**.

**Figure 1** presents the flow chart based on the PRISMA statement (Moher et al., 2009). A total of 930 abstracts were initially screened. Considering full-text availability and exclusion criteria, the final sample comprised 548 full texts that referred to the measurement of methodological quality in primary studies, using different procedures (Supplementary Data 1). Four were scales, 425 checklists, and 119 sets of general recommendations (Supplementary Table S1). The inter-rater reliability gave a κ = 0.874 (*p* < 0.001), 95% CI [0.827, 0.921].

We gathered a list of the most frequent 43-items to measure methodological quality. Supplementary Tables S2 and S3 list these items, along with the corresponding original references from Supplementary Data 1. The inter-rater reliability coefficient was κ = 0.924 (*p* < 0.001), 95% CI [0.918, 0.93]. This was considered an adequate level of agreement between the two researchers.

Finally, the 43-items identified were assigned to the previously defined dimensions and sub-dimensions according to their content (see Supplementary Table S4). Specifically, six items were assigned to extrinsic characteristics, 14 to substantive characteristics (five referred to the sample, three to the setting, and six to the intervention), and 23 to methodological characteristics. The degree of consensus across items assigned to different dimensions yielded a good agreement with a κ = 0.842 (*p* < 0.001), 95% CI [0.695, 0.989].

## Study 2. Content Validity Study

### Method

#### Sample

Thirty judges participated in the content validity study. They were experts in design, systematic reviews, quality measurement, program evaluation, and/or applied psychology (social, educational, developmental, or clinical). They were all members of the Methods Group of the Campbell Collaboration and/or European Association of Methodology. Specifically, they consisted of 12 women and 18 men, 20 from Europe and 10 from the USA. Their mean age was 42 years. They had an average of 14 years of experience on these issues.

#### Instruments

The 43-items previously obtained and structured by the dimensions were presented as a questionnaire (see Supplementary Table S4). Experts had to score each item by taking into account the three previously mentioned problems: R, U, and F (Chacón-Moscoso et al., 2001; Martínez-Arias et al., 2006). This was done using a three-point rating scale (Osterlind, 1998): -1 was the lowest, 0 the medium, and +1 the highest score. The experts could also offer suggestions (such as including another item not currently considered, modifying or eliminating existing items, or changing the dimension to which an item was assigned).

#### Procedure

##### Tool distribution and gathering

The questionnaire was sent by e-mail to 52 experts. After the third request, a total of 30 questionnaires were completed and returned. Anonymity was assured in all cases.

##### Data analysis

The Osterlind index of congruence (1998) was used to quantify the consensus between experts in their judgments of each item and issue (Glück et al., 2015). The formula used was

$$I_{ik}=\frac{(N-1)\sum_{j-1}^{n}X_{ijk}+N\sum_{j=1}^{n}X_{ijk}-\sum_{j=1}^{n}X_{ijk}}{2(N-1)n}$$

where *N* = number of dimensions; *X_ijk_* = score given by each expert to each item (between -1 and +1); and *n* = number of experts.

The results could range from -1 to +1. A score of -1 meant that all the experts awarded the most negative rating to the item in question. A score of +1 indicated that they all considered that the item in question merited the highest rating.

##### Inclusion criterion

Items that obtained a score of 0.5 or more on at least two of the three issues studied (R, U, and F) were included as important indicators to take into account when studying methodological quality in primary studies (Osterlind, 1998).

### Results

**Table 1** shows the Osterlind index obtained for each item on the three issues studied: R, U, and F. Fourteen methodological items fulfilled the inclusion criterion. A total of 18-items obtained scores equal to or higher than 0.5 on R, whereas 15-items obtained this score on U and 16 on F.

**Table 1:** Osterlind indexes of representativeness (R), utility (U), and feasibility (F) obtained for the 43 items.

Extrinsic characteristics (*N* = 30)	R	U	F
(1) Type of publication	-0.2	0.4	**0.6**
(2) Year of publication	-0.4	-0.6	**0.6**
(3) Citation impact factor for the journal	-0.4	-0.2	0
(4) Raw data from the study available	-0.8	0	**0.8**
(5) Training of treatment implementers	0.4	**0.8**	0
(6) APA format	-0.2	-0.4	-0.2
**Substantive characteristics (*N* = 30)**			
**Sample**			
(7) Age (range)	0.4	0	0.4
(8). Age (mean)	**0.6**	0.467	0.4
(9) Age (standard deviation)	-0.2	-0.4	0
(10) Cultural origin	-0.2	0.2	0.2
(11) Socioeconomic level	-0.4	0	-0.2
**Setting**			
(12) Implementation context	-0.8	-0.2	0.4
(13) Intervention field	-0.2	-0.4	**0.8**
(14) Country in which study was conducted	0.2	0.4	**0.8**
**Treatment**			
(15) Theoretical orientation	0.2	-0.2	0.2
(16) Previous empirical evidence	0	-0.2	0.4
(17) Period of treatment	0.467	0.467	**1**
(18) Degree of treatment intensity	0.4	0.467	**1**
(19) Units	**0.737**	0.433	0.467
(20) Strengths and weaknesses of treatment are discussed	0.4	-0.2	0.4
**Methodological characteristics (*N* = 30)**			
(21) Inclusion and exclusion criteria for units provided	**0.6**	**0.8**	0.4
(22) Random assignment of units	**0.8**	**1**	**0.8**
(23) Methodology or design	**0.8**	**1**	**0.8**
(24) Sample size	0.367	0.467	**1**
(25) Analysis to calculate sample size	0.4	0.4	-0.4
(26) Attrition	**0.8**	**1**	0
(27) No attrition occurred	**0.6**	**0.6**	**0.6**
(28) Attrition between groups	**1**	**1**	**0.6**
(29) Exclusions after randomization	**0.8**	**1**	0.4
(30) Units studied before treatment implementation	0	0.4	0.2
(31) Follow-up period	**0.5**	**0.6**	0.2
(32) Occasions of measurement on each variable	**0.8**	**1**	**1**
(33) Measures in pre-test appear in post-test	**0.6**	**0.8**	0.4
(34) Standardized dependent variables	**0.5**	**0.8**	0.357
(35) Intervention context homogeneity	**0.6**	0.433	0.2
(36) Control techniques	**0.6**	**0.6**	-0.2
(37) Construct definition of outcome	**1**	0**.6**	-0.2
(38) Statistical methods for imputing missing data	**0.6**	**0.6**	0.4
(39) Specification of confidence intervals in statistical analysis	0.2	0.2	**0.6**
(40) Effect size value	0.2	0.4	**0.8**
(41) Effectiveness of treatment	0	0.4	**0.8**
(42) Interpretation of results	-0.2	-0.4	0.2
(43) Discussion of bias and limitations	**0.6**	0	0.4

*Items appear in abbreviated form; the whole version can be consulted in Supplemental Material 4. Scores of 0.5 or higher are printed in bold.*

Item 22 was omitted because of its redundant content and suggestions by the experts (it shared redundant information with items 21 and 36). Furthermore, items 26 and 27 were combined into a single item. Consequently, the final proposed checklist contained 12-items focused on *methodological* characteristics. Definitions of items and their coding criteria can be found in the Appendix.

## Study 3. Inter-Coder Reliability Study

### Method

#### Sample

Four coders participated in the study. Two of them (C1 and C2) were coauthors of this study (SC and SM) and two others (C3 and C4) were not. Each coder had a high level of understanding of written English and received prior training on the coding task by an expert in the topic, also a coauthor of this article (CM).

#### Instruments

The 12-items checklist resulting from the previous Studies 1 and 2 was applied. The Appendix presents the final version of the coding scheme after including the changes derived from the pilot study described in this Study 3.

Papers were found by searching 11 computerized databases to locate training programs: EBSCO Online, Medline, Serfile, CABHealth, CINAHL, PsycINFO, Econlit, ERIC, MathSci, Current Contents, and Humanities Index. Finally, we used SPSS 17.0 to calculate Cohen’s κ coefficient.

#### Procedure

First, we conducted a bibliographic search to collect articles published in the training program field. The issue was chosen by research interest. The keywords used were “evaluation,” “training programs,” and “work.” From the resulting 1,399 published journal articles, we obtained 124 after discarding (a) the duplicates (*n* = 223); (b) those that were not written in English or Spanish (*n* = 46); (c) those for which the complete text was not available (*n* = 421); or (d) where the training program was not aimed at employees to improve their professional skills (*n* = 585). Twenty-five studies (20% of the total) were randomly selected to be used in the pilot study.

C1 and C2 were trained under the supervision of one of the authors of this article (CM), an expert on the topic. The three researchers revised the coding scheme to be sure that they understood each item in the same way (Bennett et al., 1991). CM solved the questions that C1 and C2 asked. Later, as a test, C1 and C2 jointly coded one study that was not included in this research. This task was useful to clarify some discrepancies between the coders about the items and their meaning and the way to locate the information in the papers. Then, independently, they applied the checklist to the 25 studies selected. Each study was coded in an average of 15 min.

To analyze the degree of agreement on each item, Cohen’s κ (Cohen, 1960; Bechger et al., 2003; Engelhard, 2006; Nimon et al., 2012) was used for categorical items. For quantitative items (items 3–6), a correlation coefficient was calculated. When assumptions were accepted (normality Kolmogorov–Smirnov *z* with *p* > 0.05 and independence of errors Durbin–Watson *d* between 1.5 and 2.5), the Pearson correlation coefficient (*r*) was calculated; when at least one of the assumptions was violated, the Spearman correlation coefficient (ρ) was calculated.

This reliability study was replicated twice: (a) C1 and C2 applied the scale to 20 new studies (20% of the total, randomly chosen after excluding the 25 papers previously analyzed). After analyzing the results, the wording of some definitions and alternatives of the items that might have caused coding discrepancies were modified to achieve greater clarity and simplicity in the instrument; (b) C3 and C4 applied the scale to the same 20 studies. C3 and C4 received information about the research, its main characteristics, the topic it covered, the task to do, and guidelines to codify the studies. In both replications, reliability was analyzed using the same coefficients that were used in the pilot study. In addition, the reliability among the four coders in the replication phase was analyzed. For that, we calculated Cohen’s κ for categorical items and Krippendorff’s α coefficient for quantitative items 3–6 (Hayes and Krippendorff, 2007).

### Results

#### Testing Assumptions for Quantitative Items 3–6

**Table 2** presents the results obtained on the normality (Kolmogorov–Smirnov *z*) and independence of errors (Durbin–Watson *d*) assumptions for the quantitative items 3–6.

**Table 2:** Testing assumptions for quantitative items.

	Pilot study			Replication					
Item	C1 *z*	C2 *z*	*d*	C1 *z*	C2 *z*	*d*	C3 *z*	C4 *z*	*d*
(3) Attrition	0.449	**1.696^∗∗^**	1.587	0.767	0.683	**1.289**	0.683	0.757	1.633
(4) Attrition between	0.77	0.873	2.31	0.667	0.536	**2.799**	0.49	0.595	2.244
(5) Exclusions after	1.335	0.57	**0.692**	0.451	0.513	**2.974**	0.38	0.506	**2.974**
(6) Follow-up	**1.661^∗∗^**	**1.919^∗∗^**	1.768	**1.639^∗∗^**	**1.478^∗^**	2.276	**1.532^∗^**	**1.478^∗^**	1.742

*Items appear in abbreviated form; the whole and final version (after including improvements derived from the pilot study) can be consulted in the Appendix. C1–C4 = coder 1–4, respectively. z = Kolmogorov–Smirnov z to study normality assumption (accepted when p > 0.05). d = Durbin–Watson d to study independence of errors assumption (accepted when 1.5 < d < 2.5). Results that imply an assumption violation are in boldface.*

*^∗^p < 0.05; ^∗∗^p < 0.01.*

Normality and independence of errors assumptions were accepted for item 4 in the pilot study and items 3 and 4 in the replication carried out by C3 and C4. In these cases, Pearson’s *r* coefficient was calculated as inter-coder agreement value. For the rest of the situations (when at least one assumption was violated), Spearman’s ρ coefficient was obtained.

#### Inter-coder Reliability

**Table 3** shows the results obtained for each item individually. In the pilot study, we obtained a significant agreement value for seven items; only items 4 and 10 obtained an agreement value higher than 0.7; and, in general, the 95% CI amplitudes were wide, ranging from 0.376 in item 4, [0.994, -0.618] to 1.422 in item 5, [0.551, -0.871].

**Table 3:** Results of inter-coder reliability.

	Pilot study				Replication			
	C1–C2		C1–C2		C3–C4		4C	
**Items**	**Agreement**	**95% CI**	**Agreement**	**95% CI**	**Agreement**	**95% CI**	**Agreement**	**95% CI**
(1) Inclusion/exclusion criteria	^a^0.684^∗∗^	[0.292, 1]	^a^0.798^∗∗^	[0.533, 1]	^a^0.9^∗∗^	[0.71, 1]	^a^0.851^∗∗^	[0.707, 0.995]
(2) Methodology/design	^a^0.252	[-0.062, 0.566]	^a^0.861^∗∗^	[0.277, 1]	^a^1^∗∗^	[1, 1]	^a^0.931^∗∗^	[0.639, 1]
(3) Attrition	^b^0.505	[0.078, 0.898]	^b^0.653^∗^	[0.463, 0.962]	^c^0.943^∗∗^	[0.772, 0.986]	^d^0.79^∗∗^	[0.617, 0.963]
(4) Attrition between groups	^c^0.952^∗∗^	[0.618, 0.994]	^b^0.866	[0.326, 1]	^c^0.991^∗∗^	[0.629, 1]	^d^0.849^∗∗^	[0.478, 1]
(5) Exclusions after	^b^-0.206	[-0.871, 0.551]	^b^1^∗∗^	[0.137, 0.998]	^b^1^∗∗^	[0.476, 1]	^d^0.775^∗∗^	[0.306, 1]
(6) Follow-up	^b^0.522	[-0.133, 0.802]	^b^0.783^∗∗^	[0.949, 0.994]	^b^0.963^∗∗^	[0.976, 0.997]	^d^0.76^∗∗^	[0.5, 1]
(7) Occasions of measurement	^a^0.486^∗^	[0.131, 0.841]	^a^0.653^∗∗^	[0.32, 0.986]	^a^1^∗∗^	[1, 1]	^a^0.827^∗∗^	[0.66, 0.994]
(8) Pre/post measures	^a^0.592^∗∗^	[0.173, 1]	^a^0.714^∗^	[0.212, 1]	^a^1^∗∗^	[1, 1]	^a^0.73^∗∗^	[0.606, 0.854]
(9) Dependent variables	^a^0.577^∗∗^	[0.25, 0.904]	^a^0.512^∗∗^	[0.038, 0.986]	^a^0.857^∗∗^	[0.588, 1]	^a^0.745^∗∗^	[0.313, 1]
(10) Control techniques	^a^0.706^∗∗^	[0.323, 1]	^a^0.667	[0.104, 1]	^a^0.744^∗^	[0.281, 1]	^a^0.767^∗∗^	[0.192, 1]
(11) Construct definition	^a^0.047	[-0.18, 0.274]	^a^0.861^∗∗^	[0.277, 1]	^a^0.77^∗∗^	[0.271, 1]	^a^0.772^∗∗^	[0.438, 1]
(12) Imputing missing data	^a^0.571^∗^	[0.081, 1]	^a^0.5	[0.014, 0.986]	^a^1^∗∗^	[1, 1]	^a^0.841^∗∗^	[0.581, 1]

*Items appear in abbreviated form; the whole and final version (after including improvements derived from the pilot study) can be found in the Appendix. C1–C2 = reliability results between coders 1 and 2; C3–C4 = reliability results between coders 3 and 4; 4C = reliability results across the four coders combined.*

*^a^Cohen’s κ coefficient; ^b^Spearman’s ρ coefficient; ^c^Pearson’s *r* coefficient; ^d^Krippendorff’s α coefficient.*

*^∗^p < 0.05; ^∗∗^p < 0.01.*

In the replication of the reliability study carried out by C1 and C2, we obtained a significant κ value for nine items. Four of them obtained an agreement value higher than 0.8, seven of them an agreement value higher than 0.7. The highest agreement value was 1 for item 5, *Exclusions after randomization*. The lowest agreement value was 0.5 for item 12, *Statistical methods for imputing missing data*. Compared to the results in the pilot study, the level of agreement improved substantially for most of the items except for items 4, 9, 10, and 12, where it fell slightly; 95% CIs were, in general, narrower than in the pilot study but still wide, ranging in amplitude from 0.045 (item 6, [0.994, -0.949]) to 1.168 (items 2 and 11, both [1.445, -0.277]).

In the second reliability study replication, performed by C3 and C4, the agreement value was significant for all the items. Ten items obtained an agreement value higher than 0.8. The lowest value was equal to 0.744, obtained for item 10 (*Control techniques*). Five items obtained the highest agreement value (1). Compared to the results in the replication study carried out by C1 and C2, the level of agreement was higher for C3 and C4 in all the items except for item 11, where it fell slightly, although it maintained significance and had an agreement value close to 0.8. 95% CIs were in general narrower than in the pilot study but still wide in some occasions, ranging in amplitude from 0 (items 2, 7, 8, and 12, in all cases [1-1]) to 0.998 (item 11, [1.269, -0.271]).

The results obtained in reliability across the four coders were positive, with significant values in all the items, ranging in agreement values between 0.73 and 0.931; whereas some 95% CIs remained too wide, ranging in amplitude from 0.248 (item 8, [0.854, -0.606]) to 1.15 (item 10, [1.342, -0.192]).

## Discussion

In this paper, we propose a simple 12-items checklist that, when used, can contribute to enhance the methodological quality of interventions. This checklist is formed by individual methodological features that serve as indicators of quality to be taken into account when designing, implementing, or evaluating an intervention. Thus, its use does not imply obtaining a single methodological quality measure by summing the evaluation of several indicators, which is a highly criticized approach due to the inconsistent results when measuring the same studies with different methodological quality scales (Greenland and O’Rourke, 2001).

It must be asked what this checklist adds to the state of the art. Why and how is our measurement tool any different from other proposed measures that are routinely used? The first advantage is its clear, careful, and explicit process of development. First, we made an extensively updated review of all available papers referring to the measurement of methodological quality in primary studies. Second, we carried out a content validity study through expert judges. Thus, we obtained results about the congruence between checklist items with respect to their R, U, and F in relation to the dimensions they were assigned to Osterlind (1998). Third, we carried out an inter-coder reliability pilot study and multiple replication studies. As a result, we obtained appropriate coefficients in all the items, comparing the degree of agreement in pairs and with four coders joined.

In this sense, lack of R can be considered solved. In contrast to existing publications, we have clarified to the reader how and why the checklist was developed, setting up the criteria for the inclusion of items. In this regard, the appraisal made by each item on the complete checklist can be consulted with respect to its R, U, and F; as well as in relation to the categorization of the moderator variables (i.e., substantive —about subjects, setting, and intervention—, methodological and extrinsic characteristics) usually used in a meta-analysis (Lipsey, 1994). The following information has also been made available as supplementary material: the complete list of 548 reviewed papers referring to the measurement of methodological quality in primary studies and published until July 2015 (Supplementary Data 1); the list of references classified according to different and specific approaches to the empirical definition of methodological quality (Supplementary Table S1); the 43-items chosen and the original references in which they were found (Supplementary Tables S2 and S3); and the content validity questionnaire given to experts (Supplementary Table S4).

Referring to the lack of U, some issues have been solved. The proposed 12-items checklist can be useful, not just for improving the reporting of studies. First, it can assess the methodological quality of studies that have already been carried out. It gives researchers guidelines regarding inclusion–exclusion criteria in a systematic review or meta-analysis. It also checks the methodological quality of included studies to facilitate conclusions about possible risk of bias in the conclusions. Additionally, the checklist items can be used as potential moderator variables in a meta-analysis (Conn and Rantz, 2003). Second, the checklist can enhance the methodological quality in ongoing interventions that are being planned, designed, or implemented. It is extensively useful because it can be applied to experimental and non-experimental studies (interventions with random assignment of participants to the different groups or without random assignment). This is a critical issue for practitioners and in practical systematic reviews and meta-analyses because the latter type of design is frequently used in the social sciences (Shadish et al., 2005; Mayer et al., 2014).

One advantage of focusing on methodological characteristics is that it enables the tool to be extrapolated and generalized to different areas of intervention rather than being linked to one specific context. It is therefore interesting to use a common methodological framework through which one can obtain and analyze differences and communalities both within and between different intervention contexts. Logically, conclusions obtained with the same checklist would be modulated, depending on the area of intervention.

In a parallel way, we made explicit the criteria by which we included some concrete items and excluded others. Thus, we provided practitioners and researchers with clear criteria for choosing items that may be adequate to their needs. As a consequence, some of the 43-items categorized in the extrinsic, substantive, and methodological characteristics (available in Supplementary Table S4), which were obtained from the search described in Study 1, can be selected in case researchers and practitioners are interested in including different characteristics based on their aims and specific contexts.

Referring to the lack of F, we also made advances due to the acceptable results yielded in the inter-coder reliability study (Study 3), that is, few discrepancies when different professionals coded the same studies, and because the average time needed to apply the checklist was 15 min per primary study. These facts can be interpreted in that the checklist is relatively easy to apply by having the definitions of the 12-items and their coding criteria for the final proposed checklist (Appendix).

Although this is not particularly relevant for reliability studies, the performance in Study 3 in only one intervention area is another possible limitation. Nevertheless, we are certain that the results can be generalized to other areas. We applied previous versions of the final proposed checklist in a number of pilot studies, systematic reviews, and meta-analyses. The topic was varied: psychological interventions in general, for elderly people, and for children with attention deficit hyper-activity disorder (e.g., see Supplementary Table S5). In all these cases, results obtained in inter-coder reliability were adequate.

Some of the research is ongoing or being planned. We will carry out another inter-coder reliability study enlarging the sample size to improve the accuracy of the results found in Study 3. Furthermore, we will conduct pilot studies to analyze the psychometric properties of the 12 previously obtained items. Thus, for example, we will calculate their capacity for discrimination by using the mean discrimination index and item reliability according to classical test theory (Holgado-Tello et al., 2006). Finally, the inter-coder reliability obtained was adequate but could be improved. This is why we will constantly review the definition of the 12-items of the checklist based on comments obtained from different professionals who use this tool.

## Conclusion

There is no single approach for the issue of methodological quality, and this paper was not intended to give a definitive answer. However, we do offer a justified response to the question. For that, we summarized our continuous and collaborative research over the past 15 years, which began with our first pilot applications in Baltimore in 2002 (Methods Campbell Collaboration Meeting). Furthermore, we do not merely argue the case for our own 12-items approach but also encourage other possible answers by researchers and practitioners, based on the R, U, and F assessment of the 43 most used methodological quality items in a meta-analysis.

In sum, this paper describes the rigorous process of methodological quality index selection for meta-analyses and systematic reviews and for designing, implementing, and evaluating interventions. To achieve this, we carry out an updated review on an ongoing basis. Instead of partial reviews, with poorly specified criteria for the inclusion of items, we present a checklist that has been and is being reviewed periodically. This checklist is based on the literature, experts’ opinion, applications, and feedback from related professional meetings, mainly from the *Campbell Collaboration* group (C2), the *Society for Research Synthesis Methodology* (SRSM), the *European Association of Methodology* (EAM) and the *Spanish Association of Methodology in Behavioral Sciences* (AEMCCO). The most recent comments on this work were received from the last editions of some of these meetings: the VI European Congress of Methodology in Utrecht, Netherlands (July 2014), and the XIV Congress of Methodology in Health and Social Sciences in Palma de Mallorca, Spain (July 2015).

Finally, we would like to invite any interested readers who design, implement, and/or evaluate interventions to collaborate with this project, so that we can share comments or results regarding the application of the proposed checklist. We also invite collaborations from those who are able and willing to assess the methodological quality of primary studies in meta-analyses and systematic reviews.

## Author Contributions

SC-M developed the initial idea and design of the work and performed the analysis. SS-C, and MS-M performed the analyses and interpreted the data. SC-M and SS-C were in charge of drafting the manuscript. MS-M revised the manuscript critically for important intellectual content. All three authors (SC-M, SS-C, and MS-M) provided final approval of the version to be published and agree to be accountable for all aspects of the work in ensuring that questions related to the accuracy or integrity of any part of the work were appropriately investigated and resolved.

## Conflict of Interest Statement

The authors declare that the research was conducted in the absence of any commercial or financial relationships that could be construed as a potential conflict of interest.

## Footnotes

1 www.pedro.org.au
