# Supplementary material for: The Development of a Checklist to Enhance Methodological Quality in Intervention Programs
Source: Front Psychol. 2016 Nov 18;7:1811. doi: 10.3389/fpsyg.2016.01811 (PMC5114299; doi:10.3389/fpsyg.2016.01811)
Supplement: Supplementary file 1 [file Table_1.PDF]

Supplementary Material

The development of a checklist to enhance methodological quality in intervention programs

Supplementary Table 1. List of references classified according to different and specific approaches to the empirical definition of methodological quality

APPROACH		REFERENCE
SCALE (n = 4)	Global Index (n = 2)	Classen et al. (2008); Jadad et al. (1996)
	Various Indexes (n = 2)	Cluzeau et al. (2003); Oxman and Guyatt (1991)
CHECKLIST (n = 425)	Global Index (n = 88)	Anonychuk et al. (2008); Ariens et al. (2000); Auperin et al. (1997) ; Balshem et al. (2011); Baranowsky et al. (2009); Barnes and Bero (1998); Barrat et al. (1999); Briss et al. (2000); Brown (1991); Burns and O'Connor (2008); Carayol et al. (2010); Carruthers et al. (1993); Cerin et al. (2009); Chalmers et al. (1981); Chávez (2011); Chiou et al. (2003); Cluzeau et al. (1999); Coleman et al. (2000); Coleman et al. (2013); Cornelius et al. (2011); Corrao et al. (1999); Coull and Morris (2011); de Vet et al. (1997); Declercq et al. (2011); Dennis and Dowswell (2013); Easterbrook et al. (1991); Effective Public Health Practice Project (1998); Efficace et al. (2003); El Baz et al. (2007); Espada et al. (2015); Fernández-de-las-Peñas et al. (2006); Franche et al. (2005); Garbutt et al. (1999); Gehling et al. (2011); Gerbert et al. (2011); Groenwold et al. (2008); Haynes et al. (2010); Higgins and Altman (2008); Hillberg et al. (2011); Jarde et al. (2013); Jefferson et al. (2009); Jiménez-Requena et al. (2009); Kennedy, Amick, et al. (2010); Kennedy, Medley, et al. (2010); Kleijnen et al. (1991); Kmet et al. (2004); Koricheva and Gurevitch (2014); Kung et al. (2010); Kunz and Oxman (1998); Lubans et al. (2008); MacDermid (2004); Manterola et al. (2009); Mayer et al. (2010); McGilloway et al. (2010); McNeely et al. (2006); Mistiaen and van Halm-Walters (2010); Moberg-Mogren and Nelson (2006); Moher, Fortin, et al. (1996); Moher et al. (1998); Moncrieff et al. (2001); Muche-Borowski et al. (2010); Nellensteijn et al. (2009); Newhouse et al. (2011); Olivares et al. (2000); Palermo et al. (2010); Pluye et al. (2009); Pretlove et

		al. (2006); Priebe et al. (2011); Rangel et al. (2003); Segal et al. (2003); Siddiqui et al. (2010); Slater et al. (2011); Sockol (2015); Spinewine et al. (2013); Steuten et al. (2004); Strom et al. (2014); Sutton et al. (2000); Ter Riet et al. (1990); Thomas et al. (2004); Timmer et al. (2003); Tompa et al. (2010); Tullar et al. (2010); van Abbema et al. (2011); van der Heijden et al. (1996); van Tulder et al. (1997); Yajun et al. (2010); Zhang, Lu, et al. (2013); Zhang, Sun, et al. (2013)	
	Individual components (n = 337)	1 (n = 5)	Dickersin et al. (1994); Gilbody et al. (2007); Joubert et al. (2011); Lipsey and Wilson (2001); McGrath and Degenhardt (2009)
		>1 (n = 332)	Altman et al. (2001); American Psychological Association (2010); Angelillo and Villari (1999); APA Publications and Communications Board Working Group on Journal Article Reporting Standards (2008); Arroll et al. (1988); Ashrafian and Athanasiou (2010); Baker et al. (2010); Banovac et al. (2010); Begg, et al. (1996); Beller et al. (2013); Benchimol et al. (2011); Berger et al. (2008); Berger et al. (2009); Bezerra et al. (2012); Boccia et al. (2010); Bookman et al. (2006); Booth (2006); Bosch et al. (2003); Bossuyt et al. (2003); Bousquet et al. (2009); Boutron et al. (2008); Brozek et al. (2008); Brunetti et al. (2013); Bruns (1997); Bucher et al. (1999); Burton and Altman (2004); Burton et al. (2010); Callstrom et al. (2009); Calvert et al. (2013); Campbell et al. (2004); Campbell et al. (2012); Cecile et al. (2011); Chacón et al. (2013); Chan et al. (2013); Chang et al. (2007); Chang et al. (2005); Cheng et al. (2012); Clark (2003); Clark et al. (2009); Colbert et al. (2011); Conn et al. (2011); Cook et al. (2007); Cook et al. (1992); Cornelius et al. (2009); Craig et al. (2013); Crawford et al. (2013); Critical Appraisal Skills Programme (2013); Currow et al. (2012); Dans et al. (1998); Darcourt et al. (2010); Davidoff et al. (2008); Davidson et al. (2003); Davis et al. (2011); de Keizer et al. (2012); de Vries et al. (2006); Dean et al. (2006); Dechartres et al. (2009); Delgado-Bolton et al. (2003); Department of Clinical Epidemiology and Biostatistics, McMaster University Health Sciences Centre (1981); Des Jarlais et al. (2004); Desmidt et al. (2011); Desplenter et al. (2006); Detsky et al. (1992); DeVivo et al. (2011); Dixon et al. (2010); Dobbins et al. (2004); Donahue et al. (2003); Donegan et al. (2010); Downing et al.

			(2007); Downs and Black (1998); Drummond et al. (1996, 1997, 2005); Dupuy and Simon (2007); Education Group for Guidelines on Evaluation (1999); Eken (2015); Elliot et al. (1999); Esmonde et al. (2006); Evaluation and Public Health Technologies Agency (1999); Eysenbach (2004, 2011); Faggion et al. (2015); Falkingham et al. (2010); Farrar (2009); Fernandes et al. (2009); Field et al. (2014); Fisher et al. (2009, 2012); Flores and Crepaz (2004); Fox et al. (2006); Furlan et al. (2009); Gagnier, Boon, et al. (2006); Gagnier, Kienle, et al. (2013); Gallo et al. (2012); Gao and McGrath (2011); Gardner et al. (2011); Geerts et al. (2008); Glasgow et al. (2004); Goldgruber and Ahrens (2010); Goodman et al. (1994); Goossens et al. (2011); Gotzsche (1989); Gould et al. (2001); Green and Glasgow (2006); Greenhalgh, T. (1997); Grimshaw et al. (2004, 2006); Guo et al. (2007); Guyatt et al. (1997); Guyatt, Oxman, Kunz, Woodcock, Brozek, Helfand, Alonso-Coello, Glasziou, et al. (2011); Guyatt, Oxman, Santesso, et al. (2013); Guyatt, Oxman, Sultan, et al. (2013); Guyatt, Oxman, Vist, et al. (2011); Guyatt et al. (1994); Guyatt et al. (1999); Haidet et al. (2012); Hans and Hiller (2013); Harrington and Noar (2012); Harris et al. (2001); Hayden et al. (2006); Hayward et al. (1995); Heidenreich et al. (1999); Heinsman and Shadish (1996); Heitz et al. (2009); Higashida et al. (2004); Higgins and Altman (2008); Higgins et al. (2011); Higgins and Thompson (2004); Hlatky et al. (2009); Hoffmann et al. (2014); Hollander et al. (2004); Hollenbach et al. (2011); Holt et al. (2012); Holwerda et al. (2012); Hooijmans et al. (2010); Hopewell et al. (2006, 2008); Hopley et al. (2010); Huebner et al. (2000); Hundley et al. (2009); Husereau et al. (2013); Idris et al. (1996); Jackson (2010); Jaeschke et al. (1994); Janssens et al. (2011); Johnson et al. (2014); Jorgensen and Williamson (2008); Jüni et al. (2001); Jüni et al. (1999); Karmy-Jones et al. (2011); Kausch et al. (2010); Kawai et al. (2011); Kelley et al. (2003); Kelly et al. (2007); Kempen (2011); Kent et al. (1992); Khan et al. (2000); Kienle et al. (2004); Kilkenny et al. (2010); Koch et al. (2014); Kohrt et al. (2014); Kottner et al. (2011); Lagerveld et al. (2010); Langhelle et al. (2005); Lapane et al. (2013); Lassmann et al. (2011); Laupacis et al.

			(1994, 1997); Law et al. (1998); Lee and Chi (2000); Leech and Onwuegbuzie (2010); Levine et al. (1994); Liberati et al. (2009); Linde et al. (1996, 2010); Little et al. (2009); Lu et al. (2012); MacDonald et al. (2011); Macleod et al. (2009); MacPherson et al. (2002, 2010); Maharaj and Metaxa (2011); Malterud (2001); McAlister et al. (1999); McCrory et al. (1999); McShane et al. (2005); Meads and Davenport (2009); Mello et al. (2011); Metcalf et al. (2012); Meyer et al. (2013); Mijnhout et al. (2010); Mirza and Jenkins (2004); Moher et al. (1999, 2001, 2009); Möhler et al. (2012); Mokkink et al. (2009); Moore et al. (2007, 2011); Moss and Thompson (1999); Muller-Stich et al. (2015); Munday et al. (2014); National Health and Medical Research Council (2000a); National Institute for Health and Clinical Excellence (2012); Naylor and Guyatt (1996a, 1996b); Nedeltchev et al. (2010); Newman and Elbourne (2005); Newton et al. (2009); Nicholson et al. (2008); O'Brien et al. (1997); O'Cathain et al. (2008); O'Connor et al. (2010); Oliver et al. (1996); Olson et al. (2002); Ottawa Hospital Research Institute (2013); Oxman et al. (1993, 1994); Peterson et al. (2007); Piaggio et al. (2012); Pijls et al. (2011); Pinson et al. (1991); Plonsky (2014); Plonsky and Gass (2011); Poldrack et al. (2008); Popelut et al. (2010); Portell et al. (2015); Ramsey et al. (2005); Randolph et al. (1999); Rao et al. (2011); Rauch et al. (2010); Reveiz et al. (2010); Richardson et al. (1999, 2000, 2010); Richardson and Detsky (1995a, 1995b); Ricós et al. (2008); Ridgewell el al. (2010); Riley et al. (2010); Robb et al. (2011); Ross et al. (2011); Rowan and Huston (1997); Rubino and Pragnell (1999); Rubinstein et al. (2007); Rud et al. (2009); Rutherford et al. (2010); Rutjes et al. (2006); Saint-Raymond et al. (2010); Salem et al. (2011); Sánchez-Meca (1997); Sanderson et al. (2007); Sargeant et al. (2006, 2009, 2010); Sargeant and O'Connor (2014); Sarikaya et al. (2013); Scherr et al. (2011); Schulz et al. (1995, 2010); Shadish and Ragsdale (1996); Shaneyfelt et al. (1999); Shea et al. (2007, 2009); Sherman et al. (1998); Sherrington et al. (2000); Shiffman et al. (2005); Simel et al. (2008); Sindhu et al. (1997); Siontis et al. (2010); Skoetz et al. (2013); Slatkovska et al. (2010); Slim et al. (2003); Soares et al. (2012); Sorinola et al. (2004); Staquet et al. (1996); Sterne

			et al. (2009); Stevenson et al. (2014); Stock-Schroer et al. (2009); Stone and Shiffman (2002); Stone et al. (2007); Stout et al. (2009); Stroup et al. (2000); Subramanian and Simon (2010); Sweat et al. (2012); Taji et al. (2006); Talmon et al. (2009); Tam et al. (2010); Tang et al. (2010); Terwee et al. (2011); The Asilomar Working Group on Recommendations for Reporting of Clinical Trials in the Biomedical Literature (1996); The Joanna Briggs Institute (2004); Tong et al. (2007, 2012, 2014); Tooth et al. (2005); Tran et al. (2010); Turina et al. (2009); Turlik and Kushner (2000); Turner et al. (2010); Vale et al. (2007); Valentine and Cooper (2008); Verhagen et al. (1998); Vest et al. (2010); Vintzileos and Beazoglou (2004); Virués-Ortega and Moreno-Rodríguez (2008); Viswanathan et al. (2012); Vitek et al. (2010); Vlaanderen et al. (2008); Von Elm et al. (2007); Wang et al. (2007); Watt et al. (2010); Webster et al. (2011); Weijenberg et al. (2010); Weisz et al. (2000); Welch et al. (2011, 2012); Wells et al. (2009); West et al. (2002); White (2005); Whiting et al. (2003, 2011); Widmann et al. (2009); Wolfe et al. (1999); Wong et al. (2013a, 2013b); Wortman (1994); Wu et al. (2010, 2013); Zakrzewska and Lopez (2003); Zaza et al. (2000); Zhao and Bracken (2011); Zwarenstein et al. (2008)
RECOMMENDATIONS (n = 119)			Advanced Bladder Cancer (ABC) Meta-analysis Collaboration (2005); Albrecht et al. (2013); Aletaha et al. (2008); Alexander and Cabana (2010); Alexander et al. (2010); American Educational Research Association (2006); Armstrong et al. (2007); Bartlett et al. (1998); Beccari and Oliveira (2011); Berwick (2008); Blignault and Ritchie (2009); Bow et al. (2010); Braden et al. (2011); Braithwaite et al. (2004); Brazma et al. (2001); Bril et al. (1999); Brown et al. (2009); Brown et al. (2006); Canadian Task Force on the Periodic Health Examination (1979); Carlson (2011); Cheson et al. (2003); Chesson et al. (1999); Chokshi et al. (2010); Clarke (1994); Cobo et al. (2010); Comenzo et al. (2012); Conway and Lance (2010); Cook et al. (1995); Courtney (2008); Delgado-Rodrigues (2006); Docherty and Smith (1999); Dyer et al. (2010); Dzewaltowski et al. (2004); Ehliasson (2008); Erford et al. (2011); Evans et al. (1997);

	Farrington (2003); Feneck et al. (2010); Ford and Moayyedi (2009); Friedenreich (1993); Giacomini and Cook (2000a, 2000b); Goudas et al. (2000); Greenland (1994); Grimes and Schulz (1996); Gross et al. (1994); Guyatt et al. (1998, 2000); Guyatt, Cook, et al. (2008); Guyatt, Oxman, Kunz, Brozek, et al. (2011); Guyatt, Oxman, Kunz, Woodcock, Brozek, Helfand, Alonso-Coello, Falck-Ytter, et al. (2011); Guyatt, Oxman, Montori, et al. (2011); Guyatt, Oxman, Sultan et al. (2011); Guyatt, Oxman, et al. (2008); Guyatt and Rennie (1993); Guyatt et al. (1995); Guyatt, Thorlund, et al. (2013); Harbour et al. (2011); Harbour and Miller (2001); Helmhout et al. (2008); Hemminki (1981); Howick et al. (2011); Hunt et al. (2000); Hyde (2000); Ioannidis et al. (2004); Jabs (2005); Kearon et al. (2010); Lau et al. (2000); Leonardi (2006); Li et al. (2009, 2011); Lijmer et al. (1999); Linde (2009); List and Axelsson (2010); Little (2006); Marchevsky and Gupta (2011); Matthiessen (2011); Maxwell et al. (2006); McAlister et al. (2000, 2007); McGinn et al. (2000); McGuire et al. (1985); Melby et al. (2011); Minelli et al. (2007); Moher et al. (1995); Moher, Jadad, and Tugwell, 1996); Moja et al. (2005); Möller et al. (2010); Moore et al. (2014); Müller-Riemenschneider et al. (2007); National Health and Medical Research Council (2000b); O'Rourke and Detsky (1989); Olkin (1995); Oxford Centre for Evidence-Based Medicine (2009); Petrou and Gray (2011); Plonsky and Gonulal (2015); Rozin (2009); Sackett (1989); Sánchez-Meca and Ato (1989); Scher et al. (2004); Schriger (2005); Schünemann et al. (2006); Shadish (2002); Shadish and Heinsman (1997); Shekelle et al. (1999); Simon and Lewis (2011); Skapinakis and Athanasiou (2010); Stiles et al. (2010); Tritchler (1999); Valentine and McHugh (2007); Vickers et al. (2007); Wardman (2012); Wilkinson (1999); Wilson (2009); Wilson et al. (1995); Xu (2008); Yeaton et al. (1995); Zaritsky et al. (1995); Zhang et al. (2011)
