# Supplementary material for: The Development of a Checklist to Enhance Methodological Quality in Intervention Programs
Source: Front Psychol. 2016 Nov 18;7:1811. doi: 10.3389/fpsyg.2016.01811 (PMC5114299; doi:10.3389/fpsyg.2016.01811)
Supplement: Supplementary file 2 [file Table_2.PDF]

frontiers

Supplementary Material

The development of a checklist to enhance methodological quality in intervention programs

Supplementary Table 2. 22 of the most common items to measure quality in primary studies in meta-analysis and the original references in which they were found

	1. Publication	2- Year	3. Impact factor	4. Data	5. Training	6. APA format	7. Age range	8. Age (M)	9. Age (S)	10. Cultural	11. Economic	12. Context	13. Field	14. Country	15. Theoretical	16. Empirical	17. Period	18. Intensity	19. Units	20. Discussion	21. Inclusion	22. Assignment
Advanced Blader Cancer… (2005)																						*
Albrecht et al. (2013)	*				*		*	*	*	*	*	*	*	*	*		*	*	*	*		
Aletaha et al. (2008)																	*					
Alexander et al. (2010)																						
Alexander and Cabana (2010)																						
Altman et al. (2001)					*		*	*	*	*	*	*	*	*	*	*	*	*	*	*	*	*
American Educational Research… (2006)	*				*		*	*	*	*	*	*	*	*	*	*	*	*	*	*	*	*
American Psychological Association (2010)					*	*	*	*	*	*	*	*	*	*	*	*			*		*	
American Psychological Association (2008)	*		*	*	*	*	*	*	*	*	*	*	*	*	*	*	*	*	*	*	*	*

	1. Publication	2- Year	3. Impact factor	4. Data	5. Training	6. APA format	7. Age range	8. Age (M)	9. Age (S)	10. Cultural	11. Economic	12. Context	13. Field	14. Country	15. Theoretical	16. Empirical	17. Period	18. Intensity	19. Units	20. Discussion	21. Inclusion	22. Assignment
Angelillo and Villari (1999)							*	*	*	*	*							*			*	
Anonychuk et al. (2008)						*	*							*	*							
Ariens et al. (2000)							*	*	*		*					*	*	*		*	*	
Armstrong et al. (2007)					*		*	*	*	*	*	*	*	*	*							*
Arroll et al. (1988)						*						*	*	*								
Ashrafian and Athanasiou (2010)						*	*	*	*	*	*					*				*		
Auperin et al. (1997)	*						*	*	*	*	*				*	*	*	*	*	*	*	*
Baker et al. (2010)						*						*	*	*	*	*	*	*	*		*	*
Balshem et al. (2011)																						
Banovac et al. (2010)							*	*	*	*	*	*	*				*	*	*	*	*	*
Baranowsky et al. (2009)							*	*	*	*	*	*	*				*	*	*		*	*
Barnes and Bero (1998)																					*	
Barratt et al. (1999)																*					*	
Bartlett et al. (1998)																*						
Beccari and Oliveira (2011)																						
Begg et al. (1996)				*		*	*	*	*	*	*	*		*	*		*	*	*	*	*	*

	1. Publication	2- Year	3. Impact factor	4. Data	5. Training	6. APA format	7. Age range	8. Age (M)	9. Age (S)	10. Cultural	11. Economic	12. Context	13. Field	14. Country	15. Theoretical	16. Empirical	17. Period	18. Intensity	19. Units	20. Discussion	21. Inclusion	22. Assignment
Beller et al. (2013)																					*	
Benchimol et al. (2011)					*			*						*							*	
Berger et al. (2008)													*				*	*		*		
Berger et al. (2009)				*												*	*	*		*	*	*
Berwick (2008)																						
Bezerra et al. (2012)																						*
Blignault and Ritchie (2009)	*									*	*	*	*	*	*	*					*	
Boccia et al. (2010)																					*	
Bookman et al. (2006)																						
Booth (2006)	*																*				*	*
Bosch et al. (2003)				*		*						*	*	*	*	*	*	*		*		*
Bossuyt et al. (2003)				*	*		*	*	*	*	*	*	*	*	*	*	*	*	*	*	*	*
Bousquet et al. (2009)							*	*	*	*	*	*	*	*	*	*	*	*	*	*	*	*
Boutron et al. (2008)							*	*	*	*	*	*	*	*	*	*	*	*	*	*	*	*
Bow et al. (2010)																						*
Braden et al. (2011)	*																					

	1. Publication	2- Year	3. Impact factor	4. Data	5. Training	6. APA format	7. Age range	8. Age (M)	9. Age (S)	10. Cultural	11. Economic	12. Context	13. Field	14. Country	15. Theoretical	16. Empirical	17. Period	18. Intensity	19. Units	20. Discussion	21. Inclusion	22. Assignment
Braithwaite et al. (2004)													*		*				*		*	*
Brazma et al. (2001)																						
Bril et al. (1999)																	*	*				
Briss et al. (2000)																	*	*		*	*	*
Brown et al. (2009)							*	*	*	*	*									*	*	*
Brown et al. (2006)							*	*	*	*	*	*	*			*		*		*	*	
Brown (1991)							*	*	*	*	*	*	*	*			*	*	*	*	*	*
Brozek et al. (2008)																					*	*
Brunetti et al. (2013)																						
Bruns (1997)				*			*	*	*	*	*	*									*	
Bucher et al. (1999)																*						
Burns and O’Connor (2008)								*									*	*				*
Burton and Altman (2004)																					*	
Burton et al. (2010)																						*
Callstrom et al. (2009)							*	*	*	*	*										*	*
Calvert et al. (2013)							*	*	*	*	*		*	*			*	*			*	*

	1. Publication	2- Year	3. Impact factor	4. Data	5. Training	6. APA format	7. Age range	8. Age (M)	9. Age (S)	10. Cultural	11. Economic	12. Context	13. Field	14. Country	15. Theoretical	16. Empirical	17. Period	18. Intensity	19. Units	20. Discussion	21. Inclusion	22. Assignment
Campbell et al. (2004)					*		*	*	*	*	*	*	*	*	*	*	*	*	*	*	*	*
Campbell et al. (2012)				*			*	*	*	*	*		*	*	*	*	*	*		*	*	*
Canadian Task Force… (1979)																						
Carayol et al. (2010)							*	*	*	*	*											
Carlson (2011)							*	*	*	*	*											
Carruthers et al. (1993)															*	*					*	
Cecile et al. (2011)				*			*	*	*	*	*		*	*	*	*	*	*				*
Cerin et al. (2009)															*							
Chacón et al. (2013)															*	*	*	*				
Chalmers et al. (1981)	*	*	*	*	*	*	*	*	*	*	*	*	*	*	*		*	*	*		*	*
Chan et al. (2013)					*								*	*	*	*	*	*		*		
Chang et al. (2007)							*	*	*	*	*				*	*					*	
Chang et al. (2005)						*	*	*	*	*	*	*	*	*	*	*	*	*	*	*	*	*
Chávez (2011)																						
Cheng et al. (2012)																						*
Cheson et al. (2003)							*	*	*	*	*	*	*	*							*	*

	1. Publication	2- Year	3. Impact factor	4. Data	5. Training	6. APA format	7. Age range	8. Age (M)	9. Age (S)	10. Cultural	11. Economic	12. Context	13. Field	14. Country	15. Theoretical	16. Empirical	17. Period	18. Intensity	19. Units	20. Discussion	21. Inclusion	22. Assignment
Chesson et al. (1999)																						*
Chiou et al. (2003)																				*	*	*
Chokshi et al. (2010)							*	*	*	*	*	*	*	*				*			*	*
Clark (2003)				*		*	*	*	*	*	*	*	*	*	*	*					*	
Clark et al. (2009)							*	*	*	*	*						*	*			*	
Clarke (1994)	*			*			*	*	*	*	*											*
Classen et al. (2008)	*	*	*	*	*	*	*	*	*	*	*	*	*	*	*	*	*	*	*	*	*	*
Cluzeau et al. (2003)	*		*				*	*	*	*	*	*	*	*					*		*	
Cluzeau et al. (1999)							*	*	*	*	*	*	*	*	*	*					*	*
Cobo (2010)																						
Colbert et al. (2011)				*			*	*	*	*	*	*	*	*	*	*	*	*	*		*	*
Coleman et al. (2000)																	*	*	*		*	
Coleman et al. (2013)																						
Comenzo et al. (2012)															*						*	
Conn et al. (2011)	*	*						*		*	*						*	*	*	*		*
Conway and Lance (2010)																						

	1. Publication	2- Year	3. Impact factor	4. Data	5. Training	6. APA format	7. Age range	8. Age (M)	9. Age (S)	10. Cultural	11. Economic	12. Context	13. Field	14. Country	15. Theoretical	16. Empirical	17. Period	18. Intensity	19. Units	20. Discussion	21. Inclusion	22. Assignment
Cook et al. (2007)	*		*	*			*	*	*	*	*				*	*	*	*	*	*		*
Cook et al. (1992)							*	*	*	*	*									*		*
Cook et al. (1995)																					*	*
Cornelius et al. (2009)							*	*	*	*	*										*	*
Cornelius et al. (2011)							*	*	*	*	*	*	*	*	*	*	*	*	*	*	*	
Corrao et al. (1999)					*		*	*	*	*	*										*	*
Coull and Morris (2011)							*	*	*	*	*		*		*	*	*	*	*	*		*
Courtney (2008)													*	*			*	*	*			
Craig et al. (2013)	*	*	*	*	*	*						*	*	*	*	*	*	*	*	*	*	
Crawford et al. (2013)		*			*							*	*	*	*						*	*
Critical appraisal skills programme (2013)							*	*	*	*	*	*	*	*	*	*	*	*	*	*	*	*
Currow et al. (2012)							*	*	*	*	*				*	*	*	*	*	*		
Dans et al. (1998)							*	*	*	*	*											
Darcourt et al. (2010)							*	*	*							*	*	*				
Davidoff et al. (2008)			*		*		*	*	*	*	*	*	*	*	*	*	*	*	*	*		
Davidson et al. (2003)			*		*		*	*	*	*	*	*	*	*		*	*	*	*	*	*	*

	1. Publication	2- Year	3. Impact factor	4. Data	5. Training	6. APA format	7. Age range	8. Age (M)	9. Age (S)	10. Cultural	11. Economic	12. Context	13. Field	14. Country	15. Theoretical	16. Empirical	17. Period	18. Intensity	19. Units	20. Discussion	21. Inclusion	22. Assignment
Davis et al. (2011)		*		*	*		*	*	*	*	*			*	*	*	*	*	*	*	*	
De Keizer et al. (2012)						*	*	*	*	*	*	*	*	*	*	*	*	*	*	*	*	*
De Vet et al. (1997)							*	*	*	*	*						*	*		*	*	*
De Vries et al. (2006)															*	*	*	*	*	*	*	*
Dean et al. (2006)					*		*	*	*	*	*	*	*	*		*	*	*	*	*		*
Dechartres et al. (2009)					*	*																*
Declercq et al. (2011)						*																
Delgado-Bolton et al. (2003)		*					*	*	*												*	
Delgado-Rodrigues (2006)			*																		*	
Dennis and Dowswell (2013)																						*
Department of Clinical… (1981)																*	*					
Des Jarlais et al. (2004)				*	*	*	*	*	*	*	*	*	*		*	*	*	*	*	*	*	*
Desmidt et al. (2011)										*												
Desplenter et al. (2006)							*	*	*	*	*		*	*			*	*			*	*
Detsky et al. (1992)																					*	*
DeVivo et al. (2011)							*	*	*			*										

	1. Publication	2- Year	3. Impact factor	4. Data	5. Training	6. APA format	7. Age range	8. Age (M)	9. Age (S)	10. Cultural	11. Economic	12. Context	13. Field	14. Country	15. Theoretical	16. Empirical	17. Period	18. Intensity	19. Units	20. Discussion	21. Inclusion	22. Assignment
Dickersin et al. (1994)																						*
Dixon et al. (2010)				*		*						*	*	*	*	*	*	*		*	*	*
Dobbins et al. (2004)		*				*						*	*	*		*				*		
Docherty and Smith (1999)						*										*				*		
Donahue et al. (2003)							*															
Donegan et al. (2010)							*	*	*	*	*	*	*	*	*	*	*	*	*	*		
Downing et al. (2007)					*		*	*	*	*	*		*									
Downs and Black (1998)						*	*	*	*	*	*	*	*	*	*	*	*	*	*	*	*	*
Drummond et al. (1996)				*			*	*	*	*	*				*	*				*		
Drummond et al. (2005)							*	*	*	*	*	*	*	*			*					
Drummond et al. (1997)																						
Dupuy and Simon (2007)				*			*	*	*	*	*		*							*	*	
Dyer et al. (2010)																						
Dzewaltowski et al. (2004)												*	*									
Easterbrook et al. (1991)																						*
Education Group… (1999)						*	*	*	*	*	*	*	*	*	*	*				*	*	*

	1. Publication	2- Year	3. Impact factor	4. Data	5. Training	6. APA format	7. Age range	8. Age (M)	9. Age (S)	10. Cultural	11. Economic	12. Context	13. Field	14. Country	15. Theoretical	16. Empirical	17. Period	18. Intensity	19. Units	20. Discussion	21. Inclusion	22. Assignment
Effective Public… (1998)							*	*	*	*	*	*					*	*	*			*
Efficace et al. (2003)							*	*	*	*	*		*		*		*	*				
Ehliasson (2008)																						
Eken (2015)																						*
El Baz et al. (2007)							*	*	*	*	*										*	*
Elliot et al. (1999)						*	*	*	*	*	*	*	*	*		*						
Erford et al. (2011)	*																				*	*
Esmonde et al. (2006)																					*	*
Espada et al. (2015)																						*
Evaluation and Public Health… (1999)							*	*	*	*	*	*	*	*		*	*	*			*	*
Evans et al. (1997)	*	*																				*
Eysenbach (2011)	*	*		*	*						*	*	*	*	*	*	*	*			*	*
Eysenbach (2004)							*	*	*	*	*	*	*				*				*	*
Faggion et al. (2015)																					*	*
Falkingham et al. (2010)	*	*					*											*				
Farrar (2009)				*			*	*	*	*	*				*		*	*	*	*	*	*

	1. Publication	2- Year	3. Impact factor	4. Data	5. Training	6. APA format	7. Age range	8. Age (M)	9. Age (S)	10. Cultural	11. Economic	12. Context	13. Field	14. Country	15. Theoretical	16. Empirical	17. Period	18. Intensity	19. Units	20. Discussion	21. Inclusion	22. Assignment
Farrington (2003)							*	*	*	*	*	*	*	*			*	*	*	*	*	*
Feneck et al. (2010)	*			*	*		*	*	*			*	*			*	*	*	*	*		
Fernandes et al. (2009)					*																	
Fernandez-de-las-Peñas et al. (2006)					*			*	*						*	*	*	*	*	*	*	*
Field et al. (2014)													*		*	*	*	*	*	*	*	*
Fisher et al. (2012)																					*	
Fisher et al. (2009)																					*	*
Flores and Crepaz (2004)							*	*	*	*	*				*	*	*	*	*	*		*
Ford and Moayyedi (2009)				*		*																
Fox et al. (2006)													*				*	*			*	*
Franche et al. (2005)							*	*	*	*	*				*	*	*	*	*	*	*	*
Friedenreich (1993)					*		*	*	*	*	*	*	*	*		*	*	*			*	*
Furlan et al. (2009)						*											*					*
Gagnier, Boon, et al. (2006)					*								*	*	*		*	*		*		*
Gagnier, Kienle, et al. (2013)						*	*	*	*	*	*				*	*	*	*	*	*		
Gallo et al. (2012)						*					*	*	*	*	*	*	*	*	*	*	*	*

	1. Publication	2- Year	3. Impact factor	4. Data	5. Training	6. APA format	7. Age range	8. Age (M)	9. Age (S)	10. Cultural	11. Economic	12. Context	13. Field	14. Country	15. Theoretical	16. Empirical	17. Period	18. Intensity	19. Units	20. Discussion	21. Inclusion	22. Assignment
Gao and McGrath (2011)							*	*	*	*	*	*	*	*	*	*	*	*	*	*	*	
Garbutt et al. (1999)							*	*	*	*	*										*	
Gardner et al. (2011)					*	*	*	*	*	*	*	*	*	*	*	*	*	*	*	*	*	
Geerts et al. (2008)							*	*	*	*	*				*	*	*	*	*	*		*
Gehling et al. (2011)																						*
Gerber et al. (2011)				*	*		*	*	*	*	*				*	*	*	*	*	*	*	*
Giacomini and Cook (2000a)												*	*	*							*	
Giacomini and Cook (2000b)												*	*	*	*							
Gilbody et al. (2007)							*	*	*	*	*	*	*	*							*	
Glasgow et al. (2004)																						
Goldgruber and Ahrens (2010)	*	*											*	*								
Goodman et al. (1994)						*	*	*	*	*	*	*	*	*	*	*					*	*
Goossens et al. (2011)																						
Gotzsche (1989)															*	*						*
Goudas et al. (2000)															*	*	*	*	*	*		*
Gould et al. (2001)					*		*	*	*	*	*								*			*

	1. Publication	2- Year	3. Impact factor	4. Data	5. Training	6. APA format	7. Age range	8. Age (M)	9. Age (S)	10. Cultural	11. Economic	12. Context	13. Field	14. Country	15. Theoretical	16. Empirical	17. Period	18. Intensity	19. Units	20. Discussion	21. Inclusion	22. Assignment
Green and Glasgow (2006)					*		*	*	*	*	*	*	*	*	*	*	*	*	*	*	*	
Greenhalgh (1997)					*		*	*	*	*	*	*			*	*	*	*			*	*
Greenland (1994)																						
Grimes and Schulz (1996)																					*	*
Grimshaw et al. (2006)							*	*	*	*	*	*	*				*	*				*
Grimshaw et al. (2004)																						
Groenwold et al. (2008)																						*
Gross et al. (1994)																						
Guo et al. (2007)																					*	
Guyatt, Cook, et al. (2008)							*	*	*	*	*				*	*	*	*	*	*		
Guyatt et al. (1998)																						*
Guyatt et al. (2000)																						*
Guyyat et al. (1997)													*									
Guyatt, Oxman, Kunz, Brozek, et al. (2011)																						*
Guyatt, Oxman, Kunz, Woodcock, Brozek, Helfand, Alonso-Coello, Falck, et al. (2011)																						
Guyatt, Oxman, Kunz, Woodcock, Brozek, Helfand, Alonso-Coello, Glaszio, et al.							*	*	*	*	*				*	*						

	1. Publication	2- Year	3. Impact factor	4. Data	5. Training	6. APA format	7. Age range	8. Age (M)	9. Age (S)	10. Cultural	11. Economic	12. Context	13. Field	14. Country	15. Theoretical	16. Empirical	17. Period	18. Intensity	19. Units	20. Discussion	21. Inclusion	22. Assignment
(2011)																						
Guyatt, Oxman, Montori, et al. (2011)	*															*						
Guyatt, Oxman, Santesso, et al. (2013)																						
Guyatt, Oxman, Sultan, et al. (2011)																						
Guyatt, Oxman, Sultan, et al. (2013)	*																				*	*
Guyatt, Oxman, Vist, et al. (2011)																					*	*
Guyatt, Oxman, et al. (2008)																						*
Guyatt and Rennie (1993)	*					*									*	*	*	*	*	*		
Guyatt et al. (1994)			*												*	*	*	*	*	*	*	*
Guyatt et al. (1995)																						*
Guyatt et al. (1999)	*														*	*	*	*	*	*		
Guyatt, Thorlund, et al. (2013)				*																		
Haidet et al. (2012)	*				*		*	*	*	*	*	*	*	*	*	*	*	*	*	*		*
Hans and Hiller (2013)																					*	
Harbour et al. (2011)	*				*		*	*	*	*	*				*	*	*	*	*	*	*	*
Harbour and Miller (2001)	*																					*
Harrington and Noar (2012)						*									*	*	*	*	*	*		*

	1. Publication	2- Year	3. Impact factor	4. Data	5. Training	6. APA format	7. Age range	8. Age (M)	9. Age (S)	10. Cultural	11. Economic	12. Context	13. Field	14. Country	15. Theoretical	16. Empirical	17. Period	18. Intensity	19. Units	20. Discussion	21. Inclusion	22. Assignment
Harris et al. (2001)	*														*	*	*	*	*	*	*	*
Hayden et al. (2006)							*	*	*	*	*	*	*	*	*	*	*	*	*	*		*
Haynes et al. (2010)																			*			
Hayward et al. (1995)	*		*		*							*	*	*	*	*					*	
Heidenreich et al. (1999)																	*					
Heinsman and Shadish (1996)	*	*					*	*	*	*	*	*	*									*
Heitz et al. (2009)				*			*	*	*	*	*		*		*		*	*	*	*	*	*
Helmhout et al. (2008)	*				*		*	*	*	*	*	*	*	*	*	*	*	*	*	*		*
Hemminki (1981)	*	*													*	*	*	*	*	*		*
Higashida et al. (2004)					*		*	*	*	*	*				*	*	*	*	*	*	*	*
Higgins and Altman (2008)				*																*		*
Higgins et al. (2011)																					*	
Higgins and Thompson (2004)	*		*																			
Hillberg et al. (2011)	*		*		*																	
Hlatky et al. (2009)						*																
Hoffmann et al. (2014)					*	*						*		*	*	*	*	*	*	*		

	1. Publication	2- Year	3. Impact factor	4. Data	5. Training	6. APA format	7. Age range	8. Age (M)	9. Age (S)	10. Cultural	11. Economic	12. Context	13. Field	14. Country	15. Theoretical	16. Empirical	17. Period	18. Intensity	19. Units	20. Discussion	21. Inclusion	22. Assignment
Hollander et al. (2004)						*	*	*	*	*	*	*	*	*	*	*	*	*	*	*	*	
Hollenbach et al. (2011)				*		*																*
Holt et al. (2012)																			*			*
Holwerda et al. (2012)							*	*	*	*	*				*	*	*	*	*	*	*	
Hooijmans et al. (2010)							*	*	*	*	*				*	*	*	*	*	*	*	*
Hopewell et al. (2006)		*				*	*	*	*	*	*				*		*	*	*	*		*
Hopewell et al. (2008)	*					*						*	*	*	*					*	*	*
Hopley et al. (2010)							*	*	*	*	*										*	
Howick et al. (2011)	*															*						*
Huebner et al. (2000)							*	*	*	*	*				*	*	*	*	*	*	*	*
Hundley et al. (2009)					*	*	*	*	*	*	*		*	*			*	*				
Hunt et al. (2000)	*		*												*	*						
Husereau et al. (2013)						*	*	*	*	*	*	*	*	*	*	*	*	*	*	*	*	*
Hyde (2000)	*		*			*	*	*	*	*	*				*	*	*	*	*	*	*	*
Idris et al. (1996)						*	*	*	*	*	*				*	*	*	*	*	*		*
Ioannidis et al. (2004)						*									*	*		*		*		*

	1. Publication	2- Year	3. Impact factor	4. Data	5. Training	6. APA format	7. Age range	8. Age (M)	9. Age (S)	10. Cultural	11. Economic	12. Context	13. Field	14. Country	15. Theoretical	16. Empirical	17. Period	18. Intensity	19. Units	20. Discussion	21. Inclusion	22. Assignment
Jabs (2005)					*	*						*	*	*								*
Jackson (2010)						*									*	*	*	*	*	*	*	*
Jadad et al. (1996)				*									*				*	*	*		*	*
Jaeschke et al. (1994)					*	*	*	*	*	*	*										*	
Jannssens et al. (2011)				*		*						*	*	*	*	*	*	*	*	*	*	*
Jarde et al. (2013)					*	*	*	*	*	*	*	*	*	*							*	*
Jefferson et al. (2009)																						*
Jiménez-Requena et al. (2009)							*	*	*	*	*	*	*		*	*	*	*			*	*
Johnson et al. (2014)	*		*	*			*	*	*	*	*										*	*
Jorgensen and Williamson (2008)						*	*	*	*	*	*				*	*				*		*
Joubert et al. (2011)																						
Jüni et al. (2001)						*	*	*	*	*	*	*	*	*			*	*				*
Jüni et al. (1999)		*										*										*
Karmy-Jones et al. (2011)						*	*	*	*	*	*		*		*	*	*	*	*	*		
Kausch et al. (2010)	*															*				*		*
Kawai et al. (2011)																						*

	1. Publication	2- Year	3. Impact factor	4. Data	5. Training	6. APA format	7. Age range	8. Age (M)	9. Age (S)	10. Cultural	11. Economic	12. Context	13. Field	14. Country	15. Theoretical	16. Empirical	17. Period	18. Intensity	19. Units	20. Discussion	21. Inclusion	22. Assignment
Kearon et al. (2010)						*	*	*	*	*	*										*	*
Kelley et al. (2003)						*						*	*	*		*					*	*
Kelly et al. (2007)							*	*	*								*	*		*		
Kempen (2011)							*	*	*	*	*	*	*	*	*	*	*	*	*	*	*	
Kennedy, Amick, et al. (2010)															*	*	*	*	*	*	*	*
Kennedy, Medley, et al. (2010)		*																				*
Kent et al. (1992)							*	*	*	*	*				*	*					*	
Khan et al. (2000)																						*
Kleijnen et al. (1991)							*	*	*	*	*				*	*	*	*	*	*		*
Kienle et al. (2004)		*															*	*			*	
Kilkenny et al. (2010)						*						*	*	*	*	*	*	*	*			*
Kmet et al. (2004)					*		*	*	*	*	*	*	*	*							*	*
Koch et al. (2014)					*		*	*	*	*	*				*	*	*	*	*	*	*	*
Kohrt et al. (2014)							*	*	*	*	*			*	*	*	*	*	*	*	*	*
Koricheva and Gurevitch (2014)	*	*		*																	*	
Kottner et al. (2011)				*	*	*	*	*	*	*	*										*	

	1. Publication	2- Year	3. Impact factor	4. Data	5. Training	6. APA format	7. Age range	8. Age (M)	9. Age (S)	10. Cultural	11. Economic	12. Context	13. Field	14. Country	15. Theoretical	16. Empirical	17. Period	18. Intensity	19. Units	20. Discussion	21. Inclusion	22. Assignment
Kung et al. (2010)	*			*	*		*	*	*	*	*				*	*	*	*	*	*	*	*
Kunz and Oxman (1998)					*																*	
Lagervelt et al. (2010)							*	*	*	*	*										*	*
Langhelle et al. (2005)					*		*	*	*	*	*	*	*	*								
Lapane et al. (2013)							*	*	*	*	*						*					*
Lassmann et al. (2011)							*	*	*	*	*	*	*	*	*	*	*	*	*	*		
Lau et al. (2000)							*	*	*	*	*										*	*
Laupacis et al. (1997)						*																
Laupacis et al. (1994)							*	*	*	*	*						*			*	*	
Law et al. (1998)			*			*	*	*	*	*	*	*	*	*	*	*	*	*	*	*	*	*
Lee and Chi (2000)				*		*				*				*	*	*	*					*
Leech and Onwuegbuzie (2010)	*			*			*	*	*	*	*				*	*				*		
Leonardi (2006)						*																
Levine et al. (1994)																						*
Li et al. (2009)												*	*									*
Li et al. (2011)						*															*	

	1. Publication	2- Year	3. Impact factor	4. Data	5. Training	6. APA format	7. Age range	8. Age (M)	9. Age (S)	10. Cultural	11. Economic	12. Context	13. Field	14. Country	15. Theoretical	16. Empirical	17. Period	18. Intensity	19. Units	20. Discussion	21. Inclusion	22. Assignment
Liberati et al. (2009)	*	*	*	*		*	*	*	*	*	*	*	*	*	*	*	*	*	*	*	*	*
Lijmer et al. (1999)							*	*	*	*	*											*
Linde (2009)													*									
Linde et al. (2010)																						*
Linde et al. (1996)																						*
Lipsey and Wilson (2001)																					*	
List and Axelsson (2010)																						*
Little (2006)	*																*	*			*	*
Little et al. (2009)							*	*	*	*	*						*	*			*	
Lu et al. (2012)							*	*	*	*	*										*	
Lubans et al. (2008)															*							*
MacDermid (2004)					*										*	*					*	*
MacDonald et al. (2011)						*											*	*			*	*
Macleod et al. (2009)							*	*	*	*	*										*	*
MacPherson et al. (2010)					*							*	*	*	*	*	*	*	*	*		
MacPherson et al. (2002)					*		*	*	*	*	*	*	*		*	*	*	*	*			*

	1. Publication	2- Year	3. Impact factor	4. Data	5. Training	6. APA format	7. Age range	8. Age (M)	9. Age (S)	10. Cultural	11. Economic	12. Context	13. Field	14. Country	15. Theoretical	16. Empirical	17. Period	18. Intensity	19. Units	20. Discussion	21. Inclusion	22. Assignment
Maharaj and Metaxa (2011)																						*
Malterud (2001)						*	*	*	*	*	*	*	*	*	*	*				*	*	*
Manterola et al. (2009)													*								*	*
Marchevsky and Gupta (2011)	*																					*
Matthiessen (2011)																						*
Maxwell et al. (2006)															*	*	*	*	*	*		*
Mayer et al. (2010)							*	*	*	*	*								*		*	*
McAlister et al. (1999)				*								*	*			*					*	*
McAlister et al. (2007)							*	*	*	*	*				*	*	*	*	*	*	*	*
McAlister et al. (2000)							*															*
McCrory et al. (1999)						*															*	
McGilloway et al. (2010)										*												
McGinn et al. (2000)							*	*	*	*	*											
McGrath and Degenhardt (2009)																						*
McGuire et al. (1985)																						
McNeely et al. (2006)																						*

	1. Publication	2- Year	3. Impact factor	4. Data	5. Training	6. APA format	7. Age range	8. Age (M)	9. Age (S)	10. Cultural	11. Economic	12. Context	13. Field	14. Country	15. Theoretical	16. Empirical	17. Period	18. Intensity	19. Units	20. Discussion	21. Inclusion	22. Assignment
McShane et al. (2005)				*		*	*	*	*	*	*				*	*	*	*	*	*	*	*
Meads and Davenport (2009)																					*	*
Melby et al. (2011)							*	*	*	*				*							*	*
Mello et al. (2011)																						*
Metcalf et al. (2012)																						*
Meyer et al. (2013)															*	*	*	*	*	*	*	
Mijnhout et al. (2010)																						*
Minelli et al. (2007)	*												*									
Mirza and Jenkins (2004)							*	*	*	*	*										*	
Mistiaen and van Halm-Walters (2010)																	*		*			*
Moberg-Mogren and Nelson (2006)												*	*	*							*	
Moher et al. (1999)	*					*	*	*	*	*	*	*			*	*	*	*	*	*	*	*
Moher, Fortin, et al. (1996)																						*
Moher et al. (1995)	*				*		*	*	*	*	*		*			*						*
Moher, Jadad, and Tugwell (1996)					*		*	*	*	*	*				*							*
Moher et al. (2009)	*	*		*		*	*	*	*	*	*	*	*		*	*	*	*		*	*	*

	1. Publication	2- Year	3. Impact factor	4. Data	5. Training	6. APA format	7. Age range	8. Age (M)	9. Age (S)	10. Cultural	11. Economic	12. Context	13. Field	14. Country	15. Theoretical	16. Empirical	17. Period	18. Intensity	19. Units	20. Discussion	21. Inclusion	22. Assignment
Moher et al. (1998)																						*
Moher et al. (2001)				*	*		*	*	*	*	*	*	*	*	*	*				*	*	
Möhler et al. (2012)												*	*	*	*	*	*	*	*	*		
Moja et al. (2005)																					*	*
Mokkink et al. (2009)				*			*	*	*	*	*		*			*	*	*			*	
Möller et al. (2010)																						
Moncrieff et al. (2001)							*	*	*	*	*				*	*	*	*	*	*	*	*
Moore et al. (2007)																					*	*
Moore, Derry, et al. (2014)																						*
Moore et al. (2011)							*	*	*	*	*	*	*	*			*	*				
Moss and Thompson (1999)												*	*	*								
Muche-Borowski et al. (2010)																	*					
Müller-Riemenschneider et al. (2007)																						*
Muller-Stich et al. (2015)																						*
Munday et al. (2014)																						*
National Health and Medical… (2000a)	*	*					*	*	*	*	*	*	*	*							*	*

	1. Publication	2- Year	3. Impact factor	4. Data	5. Training	6. APA format	7. Age range	8. Age (M)	9. Age (S)	10. Cultural	11. Economic	12. Context	13. Field	14. Country	15. Theoretical	16. Empirical	17. Period	18. Intensity	19. Units	20. Discussion	21. Inclusion	22. Assignment
National Health and Medical… (2000b)							*	*	*	*	*										*	*
National Institute for Health… (2012)	*	*		*		*	*	*	*	*	*	*	*	*	*		*	*			*	*
Naylor and Guyatt (1996a)					*	*						*	*	*			*	*				*
Naylor and Guyatt (1996b)												*	*	*							*	
Nedeltchev et al. (2010)																						
Nellensteijn et al. (2009)							*	*	*	*	*		*				*	*			*	
Newhouse et al. (2011)												*	*	*								
Newman and Elbourne (2005)				*		*	*	*	*	*	*	*	*	*	*	*					*	
Newton et al. (2009)						*						*	*	*			*	*				*
Nicholson et al. (2008)															*							
O’Brien et al. (1997)							*	*	*	*	*											
O’Cathain et al. (2008)					*																	
O’Connor et al. (2010)						*	*								*	*	*	*	*		*	*
O’Rourke and Detsky (1989)																*					*	
Olivares et al. (2000)	*	*			*		*	*	*	*	*	*	*				*	*	*		*	*
Oliver et al. (1996)						*	*	*	*	*	*				*	*						

	1. Publication	2- Year	3. Impact factor	4. Data	5. Training	6. APA format	7. Age range	8. Age (M)	9. Age (S)	10. Cultural	11. Economic	12. Context	13. Field	14. Country	15. Theoretical	16. Empirical	17. Period	18. Intensity	19. Units	20. Discussion	21. Inclusion	22. Assignment
Olkin (1995)	*			*			*								*						*	*
Olson et al. (2002)							*			*									*		*	
Ottawa Hospital Research Institute (2013)							*	*	*	*	*											*
Oxford Centre for Evidence… (2009)																						*
Oxman et al. (1994)							*	*	*	*	*										*	*
Oxman and Guyatt (1991)						*															*	
Oxman et al. (1993)							*	*	*	*	*									*	*	*
Palermo et al. (2010)							*	*	*	*	*										*	*
Peterson et al. (2007)						*	*	*	*	*	*	*	*	*	*	*	*				*	
Petrou and Gray (2011)																			*			*
Piaggio et al. (2012)							*	*	*	*	*											*
Pijls et al. (2011)																*	*	*	*	*	*	*
Pinson et al. (1991)																					*	
Plonsky (2014)	*	*																				*
Plonsky and Gass (2011)																						*
Plonsky and Gonulal (2015)																						*

	1. Publication	2- Year	3. Impact factor	4. Data	5. Training	6. APA format	7. Age range	8. Age (M)	9. Age (S)	10. Cultural	11. Economic	12. Context	13. Field	14. Country	15. Theoretical	16. Empirical	17. Period	18. Intensity	19. Units	20. Discussion	21. Inclusion	22. Assignment
Pluye et al. (2009)				*			*	*	*	*	*	*			*							*
Poldrack et al. (2008)							*	*	*	*	*	*	*	*			*	*	*		*	
Popelut et al. (2010)																					*	
Portell et al. (2015)					*		*	*	*	*	*	*	*	*					*		*	
Pretlove et al. (2006)							*	*	*	*	*		*									*
Priebe et al. (2011)																					*	
Ramsey et al. (2005)				*			*	*	*	*	*											
Randolph et al. (1999)												*	*	*								*
Rangel et al. (2003)						*	*	*	*	*	*	*	*	*							*	*
Rao et al. (2011)																						*
Rauch et al. (2010)												*	*	*	*	*						
Reveiz et al. (2010)								*	*	*							*	*	*		*	*
Richardson et al. (2010)										*												
Richardson and Detsky (1995a)												*	*	*							*	
Richardson and Detsky (1995b)							*	*	*	*	*											
Richardson et al. (1999)							*	*	*	*	*										*	

	1. Publication	2- Year	3. Impact factor	4. Data	5. Training	6. APA format	7. Age range	8. Age (M)	9. Age (S)	10. Cultural	11. Economic	12. Context	13. Field	14. Country	15. Theoretical	16. Empirical	17. Period	18. Intensity	19. Units	20. Discussion	21. Inclusion	22. Assignment
Richardson et al. (2000)							*	*	*	*	*						*	*	*			
Ricós et al. (2008)					*								*		*					*		*
Ridgewell et al. (2010)							*	*	*													
Riley et al. (2010)																					*	
Robb et al. (2011)					*							*	*	*	*		*	*	*	*	*	
Ross et al. (2011)							*	*	*	*	*						*	*			*	*
Rowan and Huston (1997)												*	*	*	*	*					*	
Rozin (2009)															*	*						
Rubino and Pragnell (1999)							*	*	*	*	*						*	*	*	*		
Rubinstein et al. (2007)				*			*	*	*	*	*		*		*	*	*	*			*	
Rud et al. (2009)							*	*	*	*	*	*	*				*	*			*	*
Rutherford et al. (2010)							*	*	*	*	*	*	*	*			*	*	*	*	*	
Rutjes et al. (2006)					*		*	*	*	*	*		*				*	*			*	*
Sackett (1989)																						*
Saint-Raymond et al. (2010)						*	*										*	*				*
Salem et al. (2011)							*	*	*	*	*	*	*	*			*	*	*	*	*	

	1. Publication	2- Year	3. Impact factor	4. Data	5. Training	6. APA format	7. Age range	8. Age (M)	9. Age (S)	10. Cultural	11. Economic	12. Context	13. Field	14. Country	15. Theoretical	16. Empirical	17. Period	18. Intensity	19. Units	20. Discussion	21. Inclusion	22. Assignment
Sánchez-Meca (1997)	*	*			*		*	*	*	*	*	*	*	*	*		*	*	*	*	*	*
Sánchez-Meca and Ato (1989)	*				*	*	*	*	*	*	*						*	*	*	*	*	*
Sanderson et al. (2007)												*					*	*			*	*
Sargeant et al. (2009)					*	*	*	*	*	*		*	*	*			*	*	*	*	*	*
Sargeant and O’Connor (2014)																					*	
Sargeant et al. (2010)					*	*						*	*	*			*	*	*	*	*	*
Sargeant et al. (2006)					*								*		*	*			*		*	
Sarikaya et al. (2013)																					*	
Scher et al. (2004)															*	*	*	*	*	*		
Scherr et al. (2011)																						*
Schriger (2005)						*									*		*	*				*
Schulz et al. (2010)				*			*	*	*	*	*	*	*	*	*	*	*	*	*	*	*	*
Schulz et al. (1995)																						*
Schünemann et al. (2006)										*											*	
Segal et al. (2003)						*											*	*	*		*	*
Shadish (2002)					*									*	*	*	*	*	*		*	*

	1. Publication	2- Year	3. Impact factor	4. Data	5. Training	6. APA format	7. Age range	8. Age (M)	9. Age (S)	10. Cultural	11. Economic	12. Context	13. Field	14. Country	15. Theoretical	16. Empirical	17. Period	18. Intensity	19. Units	20. Discussion	21. Inclusion	22. Assignment
Shadish and Heinsman (1997)																	*	*			*	*
Shadish and Ragsdale (1996)	*																*	*			*	*
Shaneyfelt et al. (1999)				*			*	*	*	*	*	*	*		*	*	*	*	*	*		
Shea et al. (2007)	*																				*	
Shea et al. (2009)	*						*	*	*	*	*										*	
Shekelle et al. (1999)							*	*	*	*	*	*	*									
Sherman et al. (1998)																						*
Sherrington et al. (2000)																					*	*
Shiffman et al. (2005)						*	*	*	*	*	*	*	*	*								
Siddiqui et al. (2010)							*	*	*	*	*						*	*	*	*	*	
Simel et al. (2008)					*	*	*	*	*	*	*	*	*	*					*		*	
Simon and Lewis (2011)																						
Sindhu et al. (1997)																					*	*
Siontis et al. (2010)							*	*	*	*	*										*	
Skapinakis and Athanasiou (2010)																					*	*
Skoetz et al. (2013)																						*

	1. Publication	2- Year	3. Impact factor	4. Data	5. Training	6. APA format	7. Age range	8. Age (M)	9. Age (S)	10. Cultural	11. Economic	12. Context	13. Field	14. Country	15. Theoretical	16. Empirical	17. Period	18. Intensity	19. Units	20. Discussion	21. Inclusion	22. Assignment
Slater et al. (2011)																	*	*	*	*	*	*
Slatkovska et al. (2010)							*	*	*												*	*
Slim et al. (2003)				*		*														*		
Soares et al. (2012)																				*		
Sockol (2015)					*		*	*	*	*	*						*	*	*	*	*	*
Sorinola et al. (2004)						*										*	*	*	*	*	*	
Spinewine et al. (2013)																						*
Staquet et al. (1996)						*	*	*	*	*	*	*	*	*	*	*	*	*	*	*	*	*
Sterne et al. (2009)															*							*
Steuten et al. (2004)							*	*	*	*	*	*	*	*			*	*	*	*	*	*
Stiles et al. (2010)																	*	*	*	*	*	*
Stevenson et al. (2014)																					*	*
Stock-Schroer et al. (2009)						*									*	*	*	*	*		*	*
Stone and Shiffman (2002)																	*	*	*			*
Stone et al. (2007)						*	*	*	*	*	*	*	*	*	*	*			*		*	
Stout et al. (2009)							*	*	*						*						*	*

	1. Publication	2- Year	3. Impact factor	4. Data	5. Training	6. APA format	7. Age range	8. Age (M)	9. Age (S)	10. Cultural	11. Economic	12. Context	13. Field	14. Country	15. Theoretical	16. Empirical	17. Period	18. Intensity	19. Units	20. Discussion	21. Inclusion	22. Assignment
Strom et al. (2014)		*					*	*	*	*	*	*	*									*
Stroup et al. (2000)	*		*		*		*	*	*	*	*				*	*	*	*	*	*	*	
Subramanian and Simon (2010)				*		*	*	*	*	*	*						*	*	*	*	*	
Sutton et al. (2000)																						*
Sweat et al. (2012)							*	*	*	*	*										*	*
Taji et al. (2006)																						*
Talmon et al. (2009)						*	*	*	*	*	*	*	*	*	*	*					*	
Tam et al. (2010)																						*
Tang et al. (2010)				*																	*	
Ter Riet et al. (1990)			*														*	*	*	*		*
Terwee et al. (2011)							*	*	*	*	*	*	*	*	*	*	*				*	*
The Asilomar Working Group… (1996)				*		*	*	*	*	*	*				*	*	*			*	*	*
The Joanna Briggs Institute (2004)							*	*	*	*	*				*						*	*
Thomas et al. (2004)							*	*	*	*	*						*	*		*	*	*
Timmer et al. (2003)																						*
Tompa et al. (2010)			*																			

	1. Publication	2- Year	3. Impact factor	4. Data	5. Training	6. APA format	7. Age range	8. Age (M)	9. Age (S)	10. Cultural	11. Economic	12. Context	13. Field	14. Country	15. Theoretical	16. Empirical	17. Period	18. Intensity	19. Units	20. Discussion	21. Inclusion	22. Assignment
Tong et al. (2012)	*	*	*				*	*	*	*	*			*	*		*				*	
Tong et al. (2007)				*		*	*	*	*	*	*	*	*	*	*		*	*			*	
Tong et al. (2014)																						*
Tooth et al. (2005)							*	*	*	*	*	*	*	*			*				*	
Tran et al. (2010)																						
Tritchler (1999)																						*
Tullar et al. (2010)							*	*	*	*	*						*	*	*		*	*
Turina et al. (2009)							*	*	*	*	*						*	*	*	*		
Turlik and Kushner (2000)							*	*	*	*	*										*	*
Turner et al. (2010)	*					*															*	
Vale et al. (2007)				*			*	*	*	*	*	*	*		*	*			*	*		*
Valentine and Cooper (2008)		*					*	*	*	*	*	*			*		*	*	*	*	*	*
Valentine and McHugh (2007)							*	*	*	*	*						*	*			*	
van Abbema et al. (2011)							*	*	*	*	*										*	
van der Heijden et al. (1996)						*	*	*	*	*	*						*	*	*	*	*	*
van Tulder et al. (1997)							*	*	*	*	*						*	*	*	*		*

	1. Publication	2- Year	3. Impact factor	4. Data	5. Training	6. APA format	7. Age range	8. Age (M)	9. Age (S)	10. Cultural	11. Economic	12. Context	13. Field	14. Country	15. Theoretical	16. Empirical	17. Period	18. Intensity	19. Units	20. Discussion	21. Inclusion	22. Assignment
Verhagen et al. (1998)																					*	*
Vest et al. (2010)															*						*	
Vickers et al. (2007)	*															*	*	*	*			
Vintzileos and Beazoglou (2004)						*									*	*	*	*	*	*		*
Virués-Ortega and Moreno… (2008)					*	*	*	*	*	*	*	*	*	*	*	*	*	*	*		*	
Viswanathan et al. (2012)					*	*															*	*
Vitek et al. (2010)							*	*	*								*	*	*		*	
Vlaanderen et al. (2008)																	*	*				
Von Elm et al. (2007)						*	*	*	*	*	*	*	*	*	*	*	*	*	*	*	*	*
Wang et al. (2007)						*														*		
Wardman (2012)	*					*																
Watt et al. (2010)																						
Webster et al. (2011)							*	*	*	*	*										*	
Weijenberg et al. (2010)							*	*	*	*	*						*					*
Weisz et al. (2000)				*									*		*							
Welch et al. (2011)															*	*	*	*			*	*

	1. Publication	2- Year	3. Impact factor	4. Data	5. Training	6. APA format	7. Age range	8. Age (M)	9. Age (S)	10. Cultural	11. Economic	12. Context	13. Field	14. Country	15. Theoretical	16. Empirical	17. Period	18. Intensity	19. Units	20. Discussion	21. Inclusion	22. Assignment
Welch et al. (2012)	*					*						*	*	*	*						*	
Wells et al. (2009)							*	*	*	*	*		*				*	*	*		*	*
West et al. (2002)							*	*	*	*	*						*	*	*	*	*	*
White (2005)						*	*	*	*	*	*	*	*	*	*	*					*	
Whiting et al. (2003)				*			*	*	*	*	*						*	*			*	
Whiting et al. (2011)							*	*	*	*	*	*	*	*							*	*
Widmann et al. (2009)	*																					*
Wilkinson (1999)	*																					*
Wilson (2009)	*	*																				
Wilson et al. (1995)												*	*	*								*
Wolfe et al. (1999)					*	*	*	*	*	*	*										*	
Wong et al. (2013a)	*					*	*	*	*	*	*				*	*					*	
Wong et al. (2013b)						*	*	*	*	*	*				*	*					*	
Wortman (1994)	*		*	*			*	*	*	*	*	*	*	*	*					*		*
Wu et al. (2013)																						*
Wu et al. (2010)	*					*	*	*	*	*	*		*	*			*	*		*		

	1. Publication	2- Year	3. Impact factor	4. Data	5. Training	6. APA format	7. Age range	8. Age (M)	9. Age (S)	10. Cultural	11. Economic	12. Context	13. Field	14. Country	15. Theoretical	16. Empirical	17. Period	18. Intensity	19. Units	20. Discussion	21. Inclusion	22. Assignment
Xu (2008)	*	*		*			*	*	*	*	*	*		*	*						*	*
Yajun et al. (2010)							*	*	*	*	*						*	*	*			*
Yeaton et al. (1995)							*	*	*	*	*		*						*			*
Zakrzewska and Lopez (2003)				*		*	*	*	*	*	*				*	*						*
Zaritsky et al. (1995)					*		*	*	*	*	*	*	*	*	*		*	*	*			
Zaza et al. (2000)					*		*	*	*	*	*	*	*	*		*	*	*	*		*	
Zhang, Lu, et al. (2013)							*	*	*	*	*											*
Zhang, Sun, et al. (2013)							*	*	*	*	*						*	*	*	*	*	*
Zhang et al. (2011)																						
Zhao and Bracken (2011)	*		*				*	*	*	*	*										*	*
Zwarenstein et al. (2008)						*	*	*	*	*	*	*	*	*		*				*	*	

Note. References and items appear in abbreviated form. The full version can be consulted, in the same order, in Supplementary Data 1 and Supplementary Table 4 respectively.
