# Supplementary material for: The Development of a Checklist to Enhance Methodological Quality in Intervention Programs
Source: Front Psychol. 2016 Nov 18;7:1811. doi: 10.3389/fpsyg.2016.01811 (PMC5114299; doi:10.3389/fpsyg.2016.01811)
Supplement: Supplementary file 3 [file Table_3.PDF]

Supplementary Material

The development of a checklist to enhance methodological quality in intervention programs

Supplementary Table 3. The remaining 21 most common items to measure quality in primary studies in meta-analysis and the original references in which they were found

	23. Design	24. Sample size	25. Analysis	26. Attrition	27- No attrition	28. At. groups	29. Exclusions	30. Before	31. Follow-up	32. Occasions	33. Measures	34. D. V.	35. Homogeneity	36. Control	37. Construct	38. Missing data	39. C. I.	40. Effect size	41. Effectiveness	42. Interpretation	43. Limitations
Advanced Blader Cancer… (2005)									*			*			*						
Albrecht et al. (2013)	*			*	*			*		*	*		*								
Aletaha et al. (2008)									*	*	*				*						*
Alexander et al. (2010)												*		*	*						
Alexander and Cabana (2010)											*	*			*						
Altman et al. (2001)	*	*	*	*	*	*	*	*	*	*		*	*	*	*		*	*	*	*	*
American Educational Research… (2006)	*	*		*	*					*	*	*	*	*	*		*	*		*	*
American Psychological Association (2010)	*	*	*	*	*	*	*	*	*	*	*	*	*	*	*	*	*	*	*	*	*
American Psychological Association (2008)	*	*	*	*	*	*	*	*	*	*	*	*	*	*	*	*	*	*	*	*	*

	23. Design	24. Sample size	25. Analysis	26. Attrition	27- No attrition	28. At. groups	29. Exclusions	30. Before	31. Follow-up	32. Occasions	33. Measures	34. D. V.	35. Homogeneity	36. Control	37. Construct	38. Missing data	39. C. I.	40. Effect size	41. Effectiveness	42. Interpretation	43. Limitations
Angelillo and Villari (1999)	*					*	*		*			*		*	*		*	*			
Anonychuk et al. (2008)	*		*									*	*	*					*	*	*
Ariens et al. (2000)	*			*	*		*		*	*	*	*	*	*	*		*				
Armstrong et al. (2007)	*								*			*	*	*		*				*	
Arroll et al. (1988)												*			*					*	
Ashrafian and Athanasiou (2010)	*								*			*	*				*		*	*	*
Auperin et al. (1997)														*			*	*	*	*	*
Baker et al. (2010)	*	*								*			*	*	*					*	*
Balshem et al. (2011)	*													*				*	*	*	*
Banovac et al. (2010)	*	*	*							*		*				*	*	*			
Baranowsky et al. (2009)	*	*		*	*	*	*	*	*	*	*		*	*		*	*	*	*		
Barnes and Bero (1998)																				*	*
Barratt et al. (1999)																			*	*	*
Bartlett et al. (1998)	*																				
Beccari and Oliveira (2011)	*																				

	23. Design	24. Sample size	25. Analysis	26. Attrition	27- No attrition	28. At. groups	29. Exclusions	30. Before	31. Follow-up	32. Occasions	33. Measures	34. D. V.	35. Homogeneity	36. Control	37. Construct	38. Missing data	39. C. I.	40. Effect size	41. Effectiveness	42. Interpretation	43. Limitations
Begg et al. (1996)	*	*	*	*	*	*	*		*					*	*	*	*	*	*	*	*
Beller et al. (2013)	*	*															*	*	*	*	*
Benchimol et al. (2011)		*		*					*					*			*		*	*	*
Berger et al. (2008)		*												*	*						
Berger et al. (2009)	*	*						*	*	*	*	*	*	*	*				*	*	*
Berwick (2008)	*													*							*
Bezerra et al. (2012)																					
Blignault and Ritchie (2009)	*				*							*								*	*
Boccia et al. (2010)																		*			
Bookman et al. (2006)									*												
Booth (2006)	*																				
Bosch et al. (2003)	*	*	*								*	*	*		*		*	*	*	*	*
Bossuyt et al. (2003)	*	*	*	*	*	*	*	*	*	*		*		*	*	*	*	*	*	*	*
Bousquet et al. (2009)	*	*	*	*	*	*	*	*	*	*	*	*	*	*	*	*	*	*	*	*	*
Boutron et al. (2008)	*	*	*	*	*	*	*	*	*	*	*	*	*	*	*	*	*	*	*	*	*

	23. Design	24. Sample size	25. Analysis	26. Attrition	27- No attrition	28. At. groups	29. Exclusions	30. Before	31. Follow-up	32. Occasions	33. Measures	34. D. V.	35. Homogeneity	36. Control	37. Construct	38. Missing data	39. C. I.	40. Effect size	41. Effectiveness	42. Interpretation	43. Limitations
Bow et al. (2010)																					
Braden et al. (2011)																					
Braithwaite et al. (2004)	*	*	*						*	*		*	*		*		*	*	*	*	*
Brazma et al. (2001)	*																				
Bril et al. (1999)	*	*		*	*				*					*							
Briss et al. (2000)	*	*							*	*		*	*	*							*
Brown et al. (2009)	*			*	*	*				*	*	*		*					*	*	*
Brown et al. (2006)	*								*					*							
Brown (1991)	*	*	*						*	*		*			*						
Brozek et al. (2008)				*	*	*								*							
Brunetti et al. (2013)	*																				*
Bruns (1997)	*	*					*						*	*	*				*	*	*
Bucher et al. (1999)																			*		*
Burns and O’Connor (2008)									*					*							
Burton and Altman (2004)		*					*									*					

	23. Design	24. Sample size	25. Analysis	26. Attrition	27- No attrition	28. At. groups	29. Exclusions	30. Before	31. Follow-up	32. Occasions	33. Measures	34. D. V.	35. Homogeneity	36. Control	37. Construct	38. Missing data	39. C. I.	40. Effect size	41. Effectiveness	42. Interpretation	43. Limitations
Burton et al. (2010)	*			*	*	*						*		*		*					
Callstrom et al. (2009)	*								*				*					*	*	*	*
Calvert et al. (2013)	*	*	*	*	*	*						*		*				*	*	*	*
Campbell et al. (2004)	*	*	*	*	*	*	*	*	*	*	*	*	*	*	*		*	*	*	*	*
Campbell et al. (2012)	*	*		*	*	*			*					*			*	*	*	*	*
Canadian Task Force… (1979)	*																				
Carayol et al. (2010)	*											*		*						*	
Carlson (2011)	*																				
Carruthers et al. (1993)	*	*							*					*	*						
Cecile et al. (2011)	*	*							*			*				*			*	*	*
Cerin et al. (2009)	*											*						*			
Chacón et al. (2013)		*										*							*	*	*
Chalmers et al. (1981)	*	*	*	*	*	*	*	*		*			*		*		*	*	*	*	*
Chan et al. (2013)		*							*			*		*					*	*	*
Chang et al. (2007)		*		*	*	*				*			*				*		*	*	*

	23. Design	24. Sample size	25. Analysis	26. Attrition	27- No attrition	28. At. groups	29. Exclusions	30. Before	31. Follow-up	32. Occasions	33. Measures	34. D. V.	35. Homogeneity	36. Control	37. Construct	38. Missing data	39. C. I.	40. Effect size	41. Effectiveness	42. Interpretation	43. Limitations
Chang et al. (2005)	*		*	*	*	*	*	*	*			*	*	*	*	*	*	*	*		*
Chávez (2011)			*											*	*						*
Cheng et al. (2012)				*	*		*							*				*			
Cheson et al. (2003)				*	*		*		*				*		*	*	*			*	
Chesson et al. (1999)	*		*																		
Chiou et al. (2003)	*							*	*	*	*	*		*	*				*		*
Chokshi et al. (2010)	*											*		*	*			*	*	*	
Clark (2003)	*		*						*			*	*	*	*					*	*
Clark et al. (2009)	*							*	*	*	*	*		*	*				*		*
Clarke (1994)			*	*	*	*	*	*	*	*	*			*		*		*			
Classen et al. (2008)	*	*	*	*	*	*	*		*	*		*	*	*	*	*	*	*	*	*	*
Cluzeau et al. (2003)								*				*	*		*			*	*	*	*
Cluzeau et al. (1999)	*							*				*		*	*				*	*	*
Cobo (2010)	*							*	*	*	*	*			*						
Colbert et al. (2011)	*	*						*	*	*	*	*	*	*	*				*		

	23. Design	24. Sample size	25. Analysis	26. Attrition	27- No attrition	28. At. groups	29. Exclusions	30. Before	31. Follow-up	32. Occasions	33. Measures	34. D. V.	35. Homogeneity	36. Control	37. Construct	38. Missing data	39. C. I.	40. Effect size	41. Effectiveness	42. Interpretation	43. Limitations
Coleman et al. (2000)	*	*							*			*									
Coleman et al. (2013)	*		*															*			
Comenzo et al. (2012)	*	*	*					*	*						*						*
Conn et al. (2011)	*			*	*	*	*														
Conway and Lance (2010)												*									
Cook et al. (2007)	*							*	*	*	*	*			*				*	*	*
Cook et al. (1992)		*											*					*	*	*	*
Cook et al. (1995)									*				*	*	*						
Cornelius et al. (2009)	*			*	*	*								*			*	*			
Cornelius et al. (2011)	*			*	*				*	*	*	*	*			*		*	*	*	
Corrao et al. (1999)	*			*	*	*		*	*	*		*	*	*	*		*	*			
Coull and Morris (2011)				*	*	*		*	*	*		*	*	*	*	*		*			
Courtney (2008)													*								
Craig et al. (2013)	*		*	*	*				*			*	*		*		*	*	*	*	*
Crawford et al. (2013)	*	*		*	*	*	*	*	*	*	*	*	*	*							

	23. Design	24. Sample size	25. Analysis	26. Attrition	27- No attrition	28. At. groups	29. Exclusions	30. Before	31. Follow-up	32. Occasions	33. Measures	34. D. V.	35. Homogeneity	36. Control	37. Construct	38. Missing data	39. C. I.	40. Effect size	41. Effectiveness	42. Interpretation	43. Limitations
Critical appraisal skills programme (2013)	*	*	*			*	*	*	*	*	*	*	*	*	*		*	*	*	*	*
Currow et al. (2012)													*						*		
Dans et al. (1998)													*						*		
Darcourt et al. (2010)	*								*			*							*	*	*
Davidoff et al. (2008)	*	*		*	*			*		*	*	*		*				*	*	*	*
Davidson et al. (2003)		*	*					*	*	*	*	*		*	*	*	*	*	*	*	*
Davis et al. (2011)	*	*		*	*	*	*	*	*	*	*	*	*	*	*	*	*	*	*	*	*
De Keizer et al. (2012)	*	*	*	*	*		*	*				*		*	*			*	*	*	*
De Vet et al. (1997)	*	*		*	*	*	*	*	*			*		*	*	*		*	*	*	
De Vries et al. (2006)				*	*		*	*				*	*	*	*						
Dean et al. (2006)	*							*				*									*
Dechartres et al. (2009)	*											*		*	*						
Declercq et al. (2011)		*																*			
Delgado-Bolton et al. (2003)	*			*	*			*	*	*	*	*	*		*		*		*	*	*
Delgado-Rodrigues (2006)		*												*				*			

	23. Design	24. Sample size	25. Analysis	26. Attrition	27- No attrition	28. At. groups	29. Exclusions	30. Before	31. Follow-up	32. Occasions	33. Measures	34. D. V.	35. Homogeneity	36. Control	37. Construct	38. Missing data	39. C. I.	40. Effect size	41. Effectiveness	42. Interpretation	43. Limitations
Dennis and Dowswell (2013)				*	*	*	*		*					*					*		
Department of Clinical… (1981)	*																	*	*		
Des Jarlais et al. (2004)	*	*	*	*	*	*	*	*	*	*	*	*	*	*	*	*	*	*	*	*	*
Desmidt et al. (2011)															*						
Desplenter et al. (2006)	*			*	*	*			*			*		*			*	*			
Detsky et al. (1992)			*	*	*		*					*	*	*			*	*	*		
DeVivo et al. (2011)																					
Dickersin et al. (1994)	*																				
Dixon et al. (2010)	*			*	*	*	*	*	*	*	*			*	*						
Dobbins et al. (2004)																			*	*	*
Docherty and Smith (1999)																			*	*	*
Donahue et al. (2003)				*	*		*										*	*	*		
Donegan et al. (2010)								*		*	*		*				*	*	*	*	
Downing et al. (2007)												*									
Downs and Black (1998)	*	*		*	*		*		*			*		*	*		*		*	*	*

	23. Design	24. Sample size	25. Analysis	26. Attrition	27- No attrition	28. At. groups	29. Exclusions	30. Before	31. Follow-up	32. Occasions	33. Measures	34. D. V.	35. Homogeneity	36. Control	37. Construct	38. Missing data	39. C. I.	40. Effect size	41. Effectiveness	42. Interpretation	43. Limitations
Drummond et al. (1996)	*											*	*		*		*	*	*	*	*
Drummond et al. (2005)				*	*		*					*			*	*					
Drummond et al. (1997)												*			*				*	*	*
Dupuy and Simon (2007)	*			*	*		*	*		*	*			*	*		*	*	*		
Dyer et al. (2010)	*	*																			
Dzewaltowski et al. (2004)				*	*		*	*	*	*	*		*						*	*	*
Easterbrook et al. (1991)	*													*							
Education Group… (1999)	*											*							*	*	*
Effective Public… (1998)	*		*	*	*	*	*	*				*	*	*		*	*	*			
Efficace et al. (2003)								*		*		*	*		*	*	*	*	*	*	*
Ehliasson (2008)															*						
Eken (2015)				*	*		*		*				*	*		*					
El Baz et al. (2007)				*	*		*	*	*			*		*	*	*		*	*		
Elliot et al. (1999)	*											*									*
Erford et al. (2011)	*																				

	23. Design	24. Sample size	25. Analysis	26. Attrition	27- No attrition	28. At. groups	29. Exclusions	30. Before	31. Follow-up	32. Occasions	33. Measures	34. D. V.	35. Homogeneity	36. Control	37. Construct	38. Missing data	39. C. I.	40. Effect size	41. Effectiveness	42. Interpretation	43. Limitations
Esmonde et al. (2006)				*	*		*					*		*					*		
Espada et al. (2015)	*	*		*	*				*			*		*				*			
Evaluation and Public Health… (1999)	*	*		*	*	*	*		*	*	*	*	*	*	*		*	*	*	*	*
Evans et al. (1997)	*	*										*		*							
Eysenbach (2011)	*							*	*	*		*	*		*				*	*	
Eysenbach (2004)	*			*	*		*	*				*	*	*	*	*					
Faggion et al. (2015)							*							*		*					
Falkingham et al. (2010)		*		*	*	*	*	*				*									
Farrar (2009)	*		*	*	*	*	*	*		*		*		*	*		*		*		
Farrington (2003)	*	*	*	*	*	*	*	*	*	*	*	*	*	*	*	*	*	*	*		
Feneck et al. (2010)	*									*	*	*							*	*	*
Fernandes et al. (2009)		*	*	*	*				*	*		*		*	*		*	*	*		
Fernandez-de-las-Peñas et al. (2006)		*		*	*			*	*			*		*	*	*	*	*			
Field et al. (2014)	*	*		*	*	*						*	*	*	*	*			*	*	*
Fisher et al. (2012)																					

	23. Design	24. Sample size	25. Analysis	26. Attrition	27- No attrition	28. At. groups	29. Exclusions	30. Before	31. Follow-up	32. Occasions	33. Measures	34. D. V.	35. Homogeneity	36. Control	37. Construct	38. Missing data	39. C. I.	40. Effect size	41. Effectiveness	42. Interpretation	43. Limitations
Fisher et al. (2009)		*	*	*	*	*	*							*							
Flores and Crepaz (2004)	*	*		*	*	*	*	*	*	*	*			*	*	*		*			
Ford and Moayyedi (2009)									*								*	*			
Fox et al. (2006)	*	*	*					*		*		*		*	*		*	*			
Franche et al. (2005)	*			*	*	*	*		*			*		*	*						*
Friedenreich (1993)	*			*	*	*	*		*	*	*		*	*							*
Furlan et al. (2009)	*			*	*	*	*	*		*	*		*	*		*					*
Gagnier, Boon, et al. (2006)	*											*			*						
Gagnier, Kienle, et al. (2013)								*	*	*	*	*							*	*	*
Gallo et al. (2012)	*	*		*	*	*		*	*	*	*	*		*		*	*	*	*	*	*
Gao and McGrath (2011)			*				*					*		*	*	*		*	*		*
Garbutt et al. (1999)	*	*		*	*			*	*	*		*	*	*	*	*	*	*	*	*	*
Gardner et al. (2011)	*	*	*				*	*		*	*	*	*	*		*	*	*	*		*
Geerts et al. (2008)	*	*						*							*				*		
Gehling et al. (2011)			*											*							

	23. Design	24. Sample size	25. Analysis	26. Attrition	27- No attrition	28. At. groups	29. Exclusions	30. Before	31. Follow-up	32. Occasions	33. Measures	34. D. V.	35. Homogeneity	36. Control	37. Construct	38. Missing data	39. C. I.	40. Effect size	41. Effectiveness	42. Interpretation	43. Limitations
Gerber et al. (2011)		*	*	*	*	*	*	*	*	*	*	*	*	*	*	*	*	*	*	*	*
Giacomini and Cook (2000a)	*									*								*		*	
Giacomini and Cook (2000b)												*							*	*	*
Gilbody et al. (2007)														*							
Glasgow et al. (2004)				*	*	*	*		*										*	*	
Goldgruber and Ahrens (2010)	*	*																*			
Goodman et al. (1994)	*			*	*	*	*					*		*	*		*	*	*	*	*
Goossens et al. (2011)												*			*						
Gotzsche (1989)	*		*					*						*	*						
Goudas et al. (2000)	*	*												*				*			
Gould et al. (2001)	*	*										*								*	
Green and Glasgow (2006)									*				*				*	*	*	*	
Greenhalgh (1997)	*	*	*	*	*	*	*		*			*	*	*	*	*	*	*	*	*	
Greenland (1994)																	*	*			
Grimes and Schulz (1996)										*											

	23. Design	24. Sample size	25. Analysis	26. Attrition	27- No attrition	28. At. groups	29. Exclusions	30. Before	31. Follow-up	32. Occasions	33. Measures	34. D. V.	35. Homogeneity	36. Control	37. Construct	38. Missing data	39. C. I.	40. Effect size	41. Effectiveness	42. Interpretation	43. Limitations
Grimshaw et al. (2006)	*																				
Grimshaw et al. (2004)															*				*		
Groenwold et al. (2008)										*	*	*		*				*			
Gross et al. (1994)	*																				
Guo et al. (2007)				*	*			*												*	
Guyatt, Cook, et al. (2008)	*													*			*	*			
Guyatt et al. (1998)	*											*		*	*		*	*	*		
Guyatt et al. (2000)	*											*		*							
Guyyat et al. (1997)	*											*		*				*			
Guyatt, Oxman, Kunz, Brozek, et al. (2011)		*	*														*	*	*		
Guyatt, Oxman, Kunz, Woodcock, Brozek, Helfand, Alonso-Coello, Falck, et al. (2011)	*													*					*		
Guyatt, Oxman, Kunz, Woodcock, Brozek, Helfand, Alonso-Coello, Glaszio, et al. (2011)							*	*											*	*	*
Guyatt, Oxman, Montori, et al. (2011)																			*	*	
Guyatt, Oxman, Santesso, et al. (2013)	*	*															*	*	*		
Guyatt, Oxman, Sultan, et al. (2011)														*				*	*		

	23. Design	24. Sample size	25. Analysis	26. Attrition	27- No attrition	28. At. groups	29. Exclusions	30. Before	31. Follow-up	32. Occasions	33. Measures	34. D. V.	35. Homogeneity	36. Control	37. Construct	38. Missing data	39. C. I.	40. Effect size	41. Effectiveness	42. Interpretation	43. Limitations
Guyatt, Oxman, Sultan, et al. (2013)	*											*		*			*				
Guyatt, Oxman, Vist, et al. (2011)	*			*	*	*			*			*		*		*			*	*	*
Guyatt, Oxman, et al. (2008)	*																		*	*	
Guyatt and Rennie (1993)																	*	*	*	*	*
Guyatt et al. (1994)	*			*	*	*	*		*	*	*		*	*	*		*	*	*	*	*
Guyatt et al. (1995)	*													*			*	*	*		
Guyatt et al. (1999)														*	*			*	*	*	*
Guyatt, Thorlund, et al. (2013)																		*			
Haidet et al. (2012)	*	*				*		*		*	*										
Hans and Hiller (2013)		*		*	*			*	*	*	*	*					*	*			
Harbour et al. (2011)	*	*				*	*	*	*	*	*	*	*	*	*	*	*	*	*	*	*
Harbour and Miller (2001)	*													*			*				
Harrington and Noar (2012)	*							*	*	*	*	*	*		*						
Harris et al. (2001)	*	*		*	*	*	*	*				*	*	*	*	*	*	*	*	*	*
Hayden et al. (2006)	*			*	*	*	*		*			*	*	*	*				*	*	*

	23. Design	24. Sample size	25. Analysis	26. Attrition	27- No attrition	28. At. groups	29. Exclusions	30. Before	31. Follow-up	32. Occasions	33. Measures	34. D. V.	35. Homogeneity	36. Control	37. Construct	38. Missing data	39. C. I.	40. Effect size	41. Effectiveness	42. Interpretation	43. Limitations
Haynes et al. (2010)								*						*							
Hayward et al. (1995)												*			*		*	*	*	*	*
Heidenreich et al. (1999)				*	*	*	*		*			*		*	*				*		
Heinsman and Shadish (1996)	*	*		*	*	*		*		*		*	*	*			*	*			
Heitz et al. (2009)	*							*		*		*	*		*	*	*	*			*
Helmhout et al. (2008)	*												*	*							
Hemminki (1981)	*	*		*	*	*	*					*	*	*	*			*	*	*	
Higashida et al. (2004)	*							*	*	*	*	*		*	*						
Higgins and Altman (2008)	*			*	*	*								*		*					*
Higgins et al. (2011)				*	*	*	*							*					*		
Higgins and Thompson (2004)									*					*		*			*		
Hillberg et al. (2011)												*	*	*	*		*	*	*	*	*
Hlatky et al. (2009)														*	*		*	*	*	*	
Hoffmann et al. (2014)													*	*							
Hollander et al. (2004)				*	*	*	*	*	*			*			*		*	*		*	*

	23. Design	24. Sample size	25. Analysis	26. Attrition	27- No attrition	28. At. groups	29. Exclusions	30. Before	31. Follow-up	32. Occasions	33. Measures	34. D. V.	35. Homogeneity	36. Control	37. Construct	38. Missing data	39. C. I.	40. Effect size	41. Effectiveness	42. Interpretation	43. Limitations
Hollenbach et al. (2011)	*											*			*	*	*		*	*	*
Holt et al. (2012)	*			*	*	*	*								*	*			*	*	*
Holwerda et al. (2012)				*	*	*	*	*	*	*	*	*	*		*			*			
Hooijmans et al. (2010)		*	*	*	*	*	*	*	*			*		*	*						*
Hopewell et al. (2006)	*	*		*	*	*				*			*	*	*	*			*	*	*
Hopewell et al. (2008)	*	*										*		*	*		*	*	*	*	*
Hopley et al. (2010)				*	*	*	*		*	*	*	*	*	*	*	*					
Howick et al. (2011)	*					*		*				*					*		*		
Huebner et al. (2000)	*							*				*							*	*	*
Hundley et al. (2009)								*		*	*	*			*		*			*	
Hunt et al. (2000)															*					*	*
Husereau et al. (2013)	*		*									*	*		*	*	*	*	*	*	*
Hyde (2000)	*											*		*	*		*	*	*	*	*
Idris et al. (1996)		*		*	*	*	*	*	*	*	*	*		*	*		*	*	*	*	*
Ioannidis et al. (2004)	*	*		*	*	*	*	*	*	*	*	*			*	*	*	*	*	*	*

	23. Design	24. Sample size	25. Analysis	26. Attrition	27- No attrition	28. At. groups	29. Exclusions	30. Before	31. Follow-up	32. Occasions	33. Measures	34. D. V.	35. Homogeneity	36. Control	37. Construct	38. Missing data	39. C. I.	40. Effect size	41. Effectiveness	42. Interpretation	43. Limitations
Jabs (2005)	*					*			*											*	*
Jackson (2010)	*	*	*	*	*	*	*	*	*	*	*	*			*	*	*	*	*	*	*
Jadad et al. (1996)	*	*		*	*	*	*		*					*	*		*	*			*
Jaeschke et al. (1994)	*											*		*	*			*	*	*	*
Jannssens et al. (2011)	*			*	*	*	*					*		*	*	*	*	*	*	*	*
Jarde et al. (2013)	*					*	*	*	*	*	*	*	*	*	*			*		*	*
Jefferson et al. (2009)	*								*					*							
Jiménez-Requena et al. (2009)	*			*	*	*		*	*	*		*	*		*	*	*	*	*	*	
Johnson et al. (2014)	*						*							*				*		*	*
Jorgensen and Williamson (2008)	*	*	*	*	*	*	*				*	*		*	*	*	*	*	*		
Joubert et al. (2011)																	*	*			
Jüni et al. (2001)	*			*	*	*	*		*						*	*					
Jüni et al. (1999)	*													*						*	*
Karmy-Jones et al. (2011)		*						*	*	*	*	*			*						
Kausch et al. (2010)	*	*	*					*				*		*	*	*	*	*	*	*	*

	23. Design	24. Sample size	25. Analysis	26. Attrition	27- No attrition	28. At. groups	29. Exclusions	30. Before	31. Follow-up	32. Occasions	33. Measures	34. D. V.	35. Homogeneity	36. Control	37. Construct	38. Missing data	39. C. I.	40. Effect size	41. Effectiveness	42. Interpretation	43. Limitations
Kawai et al. (2011)	*			*	*	*	*		*					*							
Kearon et al. (2010)	*			*	*	*	*		*			*			*		*		*	*	*
Kelley et al. (2003)	*			*	*	*	*					*			*			*	*	*	*
Kelly et al. (2007)								*				*		*	*				*	*	*
Kempen (2011)															*		*	*		*	*
Kennedy, Amick, et al. (2010)	*			*			*	*	*	*	*		*	*	*	*		*	*		
Kennedy, Medley, et al. (2010)	*							*	*	*	*										
Kent et al. (1992)		*						*	*			*	*					*			
Khan et al. (2000)	*			*	*	*	*	*			*			*		*					
Kleijnen et al. (1991)	*					*								*			*	*			
Kienle et al. (2004)		*						*	*	*	*	*		*	*					*	*
Kilkenny et al. (2010)	*	*	*	*	*	*	*	*	*	*	*			*	*	*	*	*	*	*	*
Kmet et al. (2004)	*							*				*		*	*		*	*	*	*	*
Koch et al. (2014)	*	*	*					*						*	*			*			
Kohrt et al. (2014)	*	*	*	*	*	*	*	*	*		*	*	*	*	*	*		*			

	23. Design	24. Sample size	25. Analysis	26. Attrition	27- No attrition	28. At. groups	29. Exclusions	30. Before	31. Follow-up	32. Occasions	33. Measures	34. D. V.	35. Homogeneity	36. Control	37. Construct	38. Missing data	39. C. I.	40. Effect size	41. Effectiveness	42. Interpretation	43. Limitations
Koricheva and Gurevitch (2014)												*		*	*		*	*	*	*	
Kottner et al. (2011)		*	*					*		*	*	*		*	*		*	*	*	*	*
Kung et al. (2010)	*											*		*	*		*	*			*
Kunz and Oxman (1998)		*		*	*	*	*		*			*	*	*	*						
Lagervelt et al. (2010)	*	*		*	*	*	*	*	*	*	*	*		*	*		*	*			
Langhelle et al. (2005)								*				*		*	*						
Lapane et al. (2013)	*			*	*	*	*	*				*		*	*	*					
Lassmann et al. (2011)	*	*						*	*	*	*	*		*	*		*	*	*		
Lau et al. (2000)	*	*						*				*		*	*			*	*	*	
Laupacis et al. (1997)																			*	*	
Laupacis et al. (1994)				*	*	*	*	*	*					*			*	*		*	*
Law et al. (1998)	*	*	*	*	*	*		*	*	*	*			*	*	*		*	*	*	*
Lee and Chi (2000)	*			*	*	*	*	*	*	*	*	*		*	*	*					
Leech and Onwuegbuzie (2010)		*	*									*		*	*			*	*		*
Leonardi (2006)																					

	23. Design	24. Sample size	25. Analysis	26. Attrition	27- No attrition	28. At. groups	29. Exclusions	30. Before	31. Follow-up	32. Occasions	33. Measures	34. D. V.	35. Homogeneity	36. Control	37. Construct	38. Missing data	39. C. I.	40. Effect size	41. Effectiveness	42. Interpretation	43. Limitations
Levine et al. (1994)	*			*	*	*	*	*				*			*		*	*			*
Li et al. (2009)	*	*															*	*	*		
Li et al. (2011)														*	*		*	*		*	
Liberati et al. (2009)	*	*						*						*	*			*	*	*	*
Lijmer et al. (1999)	*							*				*		*					*	*	*
Linde (2009)																					
Linde et al. (2010)	*			*	*									*							
Linde et al. (1996)	*							*						*							
Lipsey and Wilson (2001)																					
List and Axelsson (2010)	*																				
Little (2006)	*			*			*	*	*			*		*							*
Little et al. (2009)				*	*	*	*	*				*		*							
Lu et al. (2012)												*	*	*	*						
Lubans et al. (2008)	*		*					*	*	*	*	*		*				*			
MacDermid (2004)	*		*	*	*	*	*	*	*	*	*		*	*	*	*		*		*	*

	23. Design	24. Sample size	25. Analysis	26. Attrition	27- No attrition	28. At. groups	29. Exclusions	30. Before	31. Follow-up	32. Occasions	33. Measures	34. D. V.	35. Homogeneity	36. Control	37. Construct	38. Missing data	39. C. I.	40. Effect size	41. Effectiveness	42. Interpretation	43. Limitations
MacDonald et al. (2011)	*	*		*	*	*	*					*		*	*			*			
Macleod et al. (2009)	*	*	*	*	*	*	*							*							*
MacPherson et al. (2010)	*												*		*						
MacPherson et al. (2002)	*											*		*			*	*			
Maharaj and Metaxa (2011)	*			*	*	*	*							*							
Malterud (2001)	*											*			*					*	*
Manterola et al. (2009)	*	*	*									*		*	*						
Marchevsky and Gupta (2011)	*																				
Matthiessen (2011)	*													*				*	*		
Maxwell et al. (2006)	*			*	*	*	*					*		*	*						
Mayer et al. (2010)		*								*	*	*			*		*		*		
McAlister et al. (1999)	*												*	*	*		*	*			*
McAlister et al. (2007)	*		*	*	*	*	*	*	*	*	*	*		*	*		*	*		*	
McAlister et al. (2000)	*	*		*	*	*	*	*							*		*	*			
McCrory et al. (1999)								*				*		*	*				*		*

	23. Design	24. Sample size	25. Analysis	26. Attrition	27- No attrition	28. At. groups	29. Exclusions	30. Before	31. Follow-up	32. Occasions	33. Measures	34. D. V.	35. Homogeneity	36. Control	37. Construct	38. Missing data	39. C. I.	40. Effect size	41. Effectiveness	42. Interpretation	43. Limitations
McGilloway et al. (2010)		*	*								*	*			*					*	*
McGinn et al. (2000)		*	*									*		*	*						*
McGrath and Degenhardt (2009)	*													*							
McGuire et al. (1985)																	*	*			
McNeely et al. (2006)	*			*	*	*	*							*		*					
McShane et al. (2005)	*	*	*	*	*	*	*	*	*	*	*	*		*	*	*	*	*	*	*	*
Meads and Davenport (2009)	*			*	*	*	*	*					*								
Melby et al. (2011)	*	*						*				*			*			*			
Mello et al. (2011)														*		*					
Metcalf et al. (2012)				*	*	*	*		*							*					
Meyer et al. (2013)								*				*		*	*			*	*		
Mijnhout et al. (2010)	*			*	*	*	*	*	*					*		*					
Minelli et al. (2007)		*															*	*	*		
Mirza and Jenkins (2004)		*	*	*	*	*	*					*			*			*	*	*	*
Mistiaen and van Halm-Walters (2010)				*	*	*	*			*	*	*		*		*		*	*		*

	23. Design	24. Sample size	25. Analysis	26. Attrition	27- No attrition	28. At. groups	29. Exclusions	30. Before	31. Follow-up	32. Occasions	33. Measures	34. D. V.	35. Homogeneity	36. Control	37. Construct	38. Missing data	39. C. I.	40. Effect size	41. Effectiveness	42. Interpretation	43. Limitations
Moberg-Mogren and Nelson (2006)															*						
Moher et al. (1999)	*	*		*	*	*	*	*	*	*	*	*		*			*	*		*	*
Moher, Fortin, et al. (1996)				*	*	*	*							*							
Moher et al. (1995)	*											*		*	*						
Moher, Jadad, and Tugwell (1996)	*								*			*		*	*		*	*			*
Moher et al. (2009)	*	*		*	*	*	*		*			*	*	*	*		*	*	*	*	*
Moher et al. (1998)	*			*	*	*	*							*							
Moher et al. (2001)		*	*	*	*	*	*	*	*	*	*		*	*	*	*	*	*	*	*	*
Möhler et al. (2012)	*											*	*	*	*			*			*
Moja et al. (2005)	*		*	*	*	*	*	*	*				*	*	*	*	*	*		*	
Mokkink et al. (2009)		*										*	*		*		*	*		*	
Möller et al. (2010)	*													*							
Moncrieff et al. (2001)	*	*	*	*	*	*	*	*	*			*		*	*	*	*	*	*		*
Moore et al. (2007)	*			*	*	*	*		*					*							
Moore, Derry, et al. (2014)				*	*	*	*							*							

	23. Design	24. Sample size	25. Analysis	26. Attrition	27- No attrition	28. At. groups	29. Exclusions	30. Before	31. Follow-up	32. Occasions	33. Measures	34. D. V.	35. Homogeneity	36. Control	37. Construct	38. Missing data	39. C. I.	40. Effect size	41. Effectiveness	42. Interpretation	43. Limitations
Moore et al. (2011)								*	*	*	*	*			*			*			
Moss and Thompson (1999)								*	*	*	*	*	*		*			*	*	*	*
Muche-Borowski et al. (2010)		*						*						*							
Müller-Riemenschneider et al. (2007)																					
Muller-Stich et al. (2015)			*						*			*	*	*	*			*			
Munday et al. (2014)	*						*	*				*	*	*	*			*			
National Health and Medical… (2000a)			*	*	*		*	*	*	*	*	*	*	*	*		*				*
National Health and Medical… (2000b)				*	*	*	*	*						*		*	*	*	*		
National Institute for Health… (2012)	*	*		*	*	*	*	*	*	*	*	*	*	*	*	*		*	*	*	*
Naylor and Guyatt (1996a)	*							*	*	*	*	*	*		*			*			
Naylor and Guyatt (1996b)												*		*							*
Nedeltchev et al. (2010)								*	*						*				*	*	
Nellensteijn et al. (2009)				*	*	*				*			*	*							
Newhouse et al. (2011)		*						*				*		*	*						
Newman and Elbourne (2005)	*	*	*	*	*	*	*				*	*		*	*		*	*		*	*

	23. Design	24. Sample size	25. Analysis	26. Attrition	27- No attrition	28. At. groups	29. Exclusions	30. Before	31. Follow-up	32. Occasions	33. Measures	34. D. V.	35. Homogeneity	36. Control	37. Construct	38. Missing data	39. C. I.	40. Effect size	41. Effectiveness	42. Interpretation	43. Limitations
Newton et al. (2009)	*		*					*				*		*	*		*	*	*	*	*
Nicholson et al. (2008)																			*		*
O’Brien et al. (1997)								*				*		*					*		*
O’Cathain et al. (2008)	*		*									*		*		*		*			
O’Connor et al. (2010)			*	*	*	*	*		*				*	*	*	*	*	*	*	*	*
O’Rourke and Detsky (1989)									*					*							
Olivares et al. (2000)	*	*		*	*	*			*	*	*	*	*	*							
Oliver et al. (1996)	*							*	*	*	*				*			*			*
Olkin (1995)	*	*						*	*	*	*			*			*	*			*
Olson et al. (2002)	*																				
Ottawa Hospital Research Institute (2013)	*			*	*	*	*	*	*	*	*	*		*	*	*		*	*		
Oxford Centre for Evidence… (2009)	*		*	*	*	*	*	*	*			*		*			*		*		
Oxman et al. (1994)			*	*	*	*	*	*	*			*	*	*							
Oxman and Guyatt (1991)	*											*		*				*		*	*
Oxman et al. (1993)	*		*	*	*	*	*	*	*			*	*	*	*	*			*	*	*

	23. Design	24. Sample size	25. Analysis	26. Attrition	27- No attrition	28. At. groups	29. Exclusions	30. Before	31. Follow-up	32. Occasions	33. Measures	34. D. V.	35. Homogeneity	36. Control	37. Construct	38. Missing data	39. C. I.	40. Effect size	41. Effectiveness	42. Interpretation	43. Limitations
Palermo et al. (2010)	*			*	*				*			*	*	*	*			*			
Peterson et al. (2007)	*	*	*					*	*	*		*		*	*	*	*	*			*
Petrou and Gray (2011)	*		*	*	*	*	*		*			*	*			*	*		*	*	*
Piaggio et al. (2012)	*		*									*	*		*		*	*		*	*
Pijls et al. (2011)	*	*		*	*		*	*	*			*			*						
Pinson et al. (1991)												*		*	*			*			
Plonsky (2014)	*							*				*		*				*			
Plonsky and Gass (2011)	*							*	*												
Plonsky and Gonulal (2015)	*																				
Pluye et al. (2009)	*	*	*	*	*	*				*		*		*		*	*	*		*	*
Poldrack et al. (2008)	*	*									*	*	*	*	*			*			*
Popelut et al. (2010)				*			*							*							
Portell et al. (2015)	*			*			*	*	*	*	*	*		*	*						
Pretlove et al. (2006)	*	*	*									*	*								
Priebe et al. (2011)				*	*	*	*	*	*			*		*							

	23. Design	24. Sample size	25. Analysis	26. Attrition	27- No attrition	28. At. groups	29. Exclusions	30. Before	31. Follow-up	32. Occasions	33. Measures	34. D. V.	35. Homogeneity	36. Control	37. Construct	38. Missing data	39. C. I.	40. Effect size	41. Effectiveness	42. Interpretation	43. Limitations
Ramsey et al. (2005)	*	*	*	*	*	*	*	*	*	*	*	*		*		*	*	*	*	*	*
Randolph et al. (1999)	*											*	*	*					*		*
Rangel et al. (2003)	*	*						*	*	*	*	*		*	*	*	*	*	*		*
Rao et al. (2011)	*		*	*	*	*	*		*					*							
Rauch et al. (2010)												*			*						
Reveiz et al. (2010)		*	*					*	*	*	*	*	*	*	*						
Richardson et al. (2010)																					
Richardson and Detsky (1995a)	*											*			*		*	*			
Richardson and Detsky (1995b)								*									*	*	*	*	
Richardson et al. (1999)			*	*	*	*	*	*				*					*	*			
Richardson et al. (2000)			*					*	*	*	*	*	*	*							
Ricós et al. (2008)	*									*		*	*						*		*
Ridgewell et al. (2010)	*							*				*			*						
Riley et al. (2010)		*	*	*	*	*	*	*	*	*	*	*				*		*			*
Robb et al. (2011)		*						*	*	*	*										

	23. Design	24. Sample size	25. Analysis	26. Attrition	27- No attrition	28. At. groups	29. Exclusions	30. Before	31. Follow-up	32. Occasions	33. Measures	34. D. V.	35. Homogeneity	36. Control	37. Construct	38. Missing data	39. C. I.	40. Effect size	41. Effectiveness	42. Interpretation	43. Limitations
Ross et al. (2011)	*		*	*	*		*	*				*		*		*	*	*			
Rowan and Huston (1997)	*		*									*		*	*				*	*	*
Rozin (2009)	*														*				*	*	
Rubino and Pragnell (1999)	*			*	*		*		*						*			*			
Rubinstein et al. (2007)		*	*	*	*	*	*	*		*		*	*	*		*	*	*	*	*	
Rud et al. (2009)	*	*	*	*	*	*	*			*		*	*	*		*	*	*		*	
Rutherford et al. (2010)		*						*	*	*	*	*	*					*			
Rutjes et al. (2006)	*	*	*	*	*	*				*		*	*	*			*	*	*	*	*
Sackett (1989)	*	*															*	*			
Saint-Raymond et al. (2010)		*						*	*	*	*			*						*	*
Salem et al. (2011)	*							*				*		*	*				*	*	*
Sánchez-Meca (1997)	*	*	*	*	*	*	*	*	*	*	*	*	*	*	*	*	*	*	*		
Sánchez-Meca and Ato (1989)	*	*	*	*	*	*	*	*	*	*	*	*	*	*	*	*					
Sanderson et al. (2007)	*	*	*							*		*		*			*	*			*
Sargeant et al. (2009)	*	*	*	*	*	*	*	*	*	*	*		*	*		*	*	*	*	*	*

	23. Design	24. Sample size	25. Analysis	26. Attrition	27- No attrition	28. At. groups	29. Exclusions	30. Before	31. Follow-up	32. Occasions	33. Measures	34. D. V.	35. Homogeneity	36. Control	37. Construct	38. Missing data	39. C. I.	40. Effect size	41. Effectiveness	42. Interpretation	43. Limitations
Sargeant and O'Connor (2014)														*	*			*			*
Sargeant et al. (2010)		*	*	*	*		*	*	*	*	*	*		*	*	*	*	*	*	*	*
Sargeant et al. (2006)													*		*		*	*	*	*	*
Sarikaya et al. (2013)	*	*		*	*		*		*					*		*					
Scher et al. (2004)	*							*	*	*	*	*			*	*		*	*	*	*
Scherr et al. (2011)	*			*	*		*							*		*					*
Schriger (2005)	*											*		*	*						*
Schulz et al. (2010)	*	*	*					*	*	*			*	*	*	*	*	*	*	*	*
Schulz et al. (1995)	*			*	*	*	*		*					*							
Schünemann et al. (2006)												*			*						*
Segal et al. (2003)	*																				*
Shadish (2002)	*	*		*	*	*	*			*						*	*	*		*	
Shadish and Heinsman (1997)	*	*	*	*	*	*	*			*				*			*	*			
Shadish and Ragsdale (1996)	*	*		*	*	*	*	*		*		*	*	*			*	*			
Shaneyfelt et al. (1999)	*			*	*		*	*	*	*	*	*			*				*	*	*

	23. Design	24. Sample size	25. Analysis	26. Attrition	27- No attrition	28. At. groups	29. Exclusions	30. Before	31. Follow-up	32. Occasions	33. Measures	34. D. V.	35. Homogeneity	36. Control	37. Construct	38. Missing data	39. C. I.	40. Effect size	41. Effectiveness	42. Interpretation	43. Limitations
Shea et al. (2007)	*													*				*			*
Shea et al. (2009)	*													*				*			*
Shekelle et al. (1999)														*							*
Sherman et al. (1998)	*	*	*	*	*	*	*							*			*		*		
Sherrington et al. (2000)	*			*	*	*	*	*				*		*	*	*	*	*			
Shiffman et al. (2005)	*			*	*		*					*		*	*						*
Siddiqui et al. (2010)		*		*	*			*	*	*	*	*	*	*	*	*					
Simel et al. (2008)	*			*	*	*	*	*	*	*	*	*		*	*	*	*	*			*
Simon and Lewis (2011)												*		*							
Sindhu et al. (1997)								*						*		*	*	*			
Siontis et al. (2010)																		*			
Skapinakis and Athanasiou (2010)	*			*	*				*					*		*					
Skoetz et al. (2013)														*		*					
Slater et al. (2011)		*						*	*	*	*	*			*						
Slatkovska et al. (2010)				*	*		*	*				*		*	*	*					

	23. Design	24. Sample size	25. Analysis	26. Attrition	27- No attrition	28. At. groups	29. Exclusions	30. Before	31. Follow-up	32. Occasions	33. Measures	34. D. V.	35. Homogeneity	36. Control	37. Construct	38. Missing data	39. C. I.	40. Effect size	41. Effectiveness	42. Interpretation	43. Limitations
Slim et al. (2003)	*		*									*		*	*					*	
Soares et al. (2012)								*	*	*	*	*			*			*	*	*	*
Sockol (2015)				*	*		*						*	*		*					
Sorinola et al. (2004)								*				*		*	*					*	*
Spinewine et al. (2013)			*									*		*			*				
Staquet et al. (1996)	*	*	*	*	*		*	*	*	*	*	*	*	*	*	*		*	*	*	*
Sterne et al. (2009)		*		*	*	*	*						*	*	*	*	*				*
Steuten et al. (2004)	*			*	*			*	*	*	*	*		*	*	*			*	*	
Stiles et al. (2010)	*		*					*	*	*	*	*			*		*	*	*	*	*
Stevenson et al. (2014)	*	*		*	*		*	*	*	*	*	*	*	*	*	*	*	*	*		
Stock-Schroer et al. (2009)	*			*	*	*	*					*	*	*	*		*	*	*	*	*
Stone and Shiffman (2002)	*			*	*		*	*				*	*	*		*					
Stone et al. (2007)	*	*	*					*	*	*	*	*	*	*	*	*	*	*		*	*
Stout et al. (2009)	*											*		*					*		
Strom et al. (2014)	*							*				*		*	*						*

	23. Design	24. Sample size	25. Analysis	26. Attrition	27- No attrition	28. At. groups	29. Exclusions	30. Before	31. Follow-up	32. Occasions	33. Measures	34. D. V.	35. Homogeneity	36. Control	37. Construct	38. Missing data	39. C. I.	40. Effect size	41. Effectiveness	42. Interpretation	43. Limitations
Stroup et al. (2000)	*											*		*	*		*	*			*
Subramanian and Simon (2010)			*									*	*		*	*	*	*	*	*	*
Sutton et al. (2000)	*								*					*			*	*			
Sweat et al. (2012)	*			*	*		*	*	*			*	*	*	*						
Taji et al. (2006)	*															*					
Talmon et al. (2009)	*			*	*	*	*					*			*				*	*	*
Tam et al. (2010)				*	*	*	*		*			*		*	*						
Tang et al. (2010)	*	*							*			*	*	*			*	*			
Ter Riet et al. (1990)	*	*		*	*		*	*	*			*	*	*	*			*	*		
Terwee et al. (2011)	*		*	*	*		*	*	*	*	*	*	*	*	*	*	*	*	*		*
The Asilomar Working Group… (1996)	*			*	*	*	*		*			*		*	*		*	*	*	*	*
The Joanna Briggs Institute (2004)	*											*			*				*	*	*
Thomas et al. (2004)	*			*	*	*	*					*	*	*				*			
Timmer et al. (2003)	*							*	*	*	*	*									
Tompa et al. (2010)																					*

	23. Design	24. Sample size	25. Analysis	26. Attrition	27- No attrition	28. At. groups	29. Exclusions	30. Before	31. Follow-up	32. Occasions	33. Measures	34. D. V.	35. Homogeneity	36. Control	37. Construct	38. Missing data	39. C. I.	40. Effect size	41. Effectiveness	42. Interpretation	43. Limitations
Tong et al. (2012)	*	*										*		*	*						*
Tong et al. (2007)	*	*		*	*		*	*						*							*
Tong et al. (2014)							*		*			*		*		*					
Tooth et al. (2005)		*	*	*	*	*	*	*	*			*		*	*	*		*			*
Tran et al. (2010)		*	*						*			*		*							
Tritchler (1999)	*																				
Tullar et al. (2010)	*			*	*	*	*	*	*			*		*	*			*	*		
Turina et al. (2009)	*											*		*	*			*			
Turlik and Kushner (2000)			*	*	*			*				*		*		*	*	*			
Turner et al. (2010)	*											*					*	*			
Vale et al. (2007)	*								*	*		*	*		*		*	*	*	*	*
Valentine and Cooper (2008)	*	*	*	*	*	*	*			*		*	*	*	*	*	*	*	*	*	*
Valentine and McHugh (2007)		*		*	*	*					*			*							
van Abbema et al. (2011)								*				*		*							
van der Heijden et al. (1996)		*		*	*			*	*			*		*	*			*			

	23. Design	24. Sample size	25. Analysis	26. Attrition	27- No attrition	28. At. groups	29. Exclusions	30. Before	31. Follow-up	32. Occasions	33. Measures	34. D. V.	35. Homogeneity	36. Control	37. Construct	38. Missing data	39. C. I.	40. Effect size	41. Effectiveness	42. Interpretation	43. Limitations
van Tulder et al. (1997)	*			*	*			*	*			*	*	*	*	*		*			
Verhagen et al. (1998)								*				*		*	*	*	*	*			
Vest et al. (2010)				*	*									*				*			
Vickers et al. (2007)	*	*						*													
Vintzileos and Beazoglou (2004)	*								*			*			*		*	*	*		*
Virués-Ortega and Moreno… (2008)	*			*	*		*	*	*	*	*	*		*	*		*	*	*	*	*
Viswanathan et al. (2012)	*			*	*	*	*	*	*			*	*	*	*	*	*				*
Vitek et al. (2010)	*	*		*	*	*	*	*				*		*	*				*		*
Vlaanderen et al. (2008)	*											*	*	*							
Von Elm et al. (2007)	*	*	*	*	*	*	*	*	*	*		*	*	*	*	*	*	*	*	*	*
Wang et al. (2007)	*							*	*			*	*		*			*			*
Wardman (2012)		*															*				
Watt et al. (2010)	*																				
Webster et al. (2011)			*					*	*			*		*	*		*	*			*
Weijenberg et al. (2010)				*	*	*	*		*									*	*		

	23. Design	24. Sample size	25. Analysis	26. Attrition	27- No attrition	28. At. groups	29. Exclusions	30. Before	31. Follow-up	32. Occasions	33. Measures	34. D. V.	35. Homogeneity	36. Control	37. Construct	38. Missing data	39. C. I.	40. Effect size	41. Effectiveness	42. Interpretation	43. Limitations
Weisz et al. (2000)												*					*	*	*	*	*
Welch et al. (2011)	*		*					*	*	*	*	*		*	*			*	*		*
Welch et al. (2012)															*			*			*
Wells et al. (2009)	*	*	*	*	*	*		*	*	*		*	*	*	*	*	*	*		*	
West et al. (2002)	*	*	*	*	*	*	*		*			*		*		*	*	*	*	*	*
White (2005)				*	*	*	*	*	*	*	*	*			*				*	*	*
Whiting et al. (2003)		*		*	*	*	*		*	*		*	*		*				*	*	*
Whiting et al. (2011)	*			*	*		*	*				*	*		*					*	
Widmann et al. (2009)	*							*	*	*	*	*			*		*				
Wilkinson (1999)	*																*	*			
Wilson (2009)																					
Wilson et al. (1995)	*	*							*			*			*		*		*		*
Wolfe et al. (1999)	*		*	*	*		*	*	*			*		*	*	*	*				*
Wong et al. (2013a)	*			*	*	*	*					*		*	*				*	*	*
Wong et al. (2013b)	*			*	*	*	*					*			*				*	*	*

	23. Design	24. Sample size	25. Analysis	26. Attrition	27- No attrition	28. At. groups	29. Exclusions	30. Before	31. Follow-up	32. Occasions	33. Measures	34. D. V.	35. Homogeneity	36. Control	37. Construct	38. Missing data	39. C. I.	40. Effect size	41. Effectiveness	42. Interpretation	43. Limitations
Wortman (1994)	*			*	*	*	*			*			*	*	*		*	*			
Wu et al. (2013)				*	*				*					*		*					
Wu et al. (2010)		*										*	*	*	*				*	*	*
Xu (2008)	*	*	*									*	*				*	*			*
Yajun et al. (2010)	*			*	*	*	*	*	*	*	*		*	*	*	*		*	*		
Yeaton et al. (1995)	*			*	*	*			*					*		*	*	*			
Zakrzewska and Lopez (2003)	*			*	*	*	*	*	*	*	*	*	*	*	*			*	*		*
Zaritsky et al. (1995)								*				*	*		*						
Zaza et al. (2000)	*	*	*	*	*	*	*	*	*	*	*	*		*	*		*	*	*	*	*
Zhang, Lu, et al. (2013)				*	*		*	*	*					*		*					
Zhang, Sun, et al. (2013)								*	*					*	*	*					
Zhang et al. (2011)	*																				
Zhao and Bracken (2011)	*			*	*		*							*					*		
Zwarenstein et al. (2008)								*	*			*			*					*	

Note. References and items appear in abbreviated form. The full version can be consulted, in the same order, in Supplementary Data 1 and Supplementary Table 4 respectively.
