# Supplementary material for: The Development of a Checklist to Enhance Methodological Quality in Intervention Programs
Source: Front Psychol. 2016 Nov 18;7:1811. doi: 10.3389/fpsyg.2016.01811 (PMC5114299; doi:10.3389/fpsyg.2016.01811)
Supplement: Supplementary file 4 [file Table_4.PDF]

Supplementary Material

The development of a checklist to enhance methodological quality in intervention programs

Supplementary Table 4. Content validity questionnaire

We ask for your collaboration to complete this questionnaire in order to assess the content validity of a scale to measure the quality of primary studies for meta-analysis.

Specifically, we would like you to assess each of the following items and score them as -1 (minimum level), 0 (medium level) or +1 (maximum level) with respect to their:

1. Representativeness: How much the specific item represents the quality subdomain to which it is assigned.
2. Utility: How much the specific item is useful for assessing the quality of the study with respect to the quality domain to which it is assigned.
3. Feasibility: How feasible it is to code that item (the extent to which data codification is viable because data are available and can be gathered).

For each item we also ask you for comments and to suggest any other item that you think it is important to take into account.

Thanks in advance for your time and collaboration.

		Item	Representative	Useful	Feasible	Comments
**EXTRINSIC CHARACTERISTICS**		1- Type of publication (1. Journal, 2. Book, 3. Thesis, 4. Congress, 5. Other)	(-1) (0) (1)	(-1) (0) (1)	(-1) (0) (1)	
		2- Year of publication	(-1) (0) (1)	(-1) (0) (1)	(-1) (0) (1)	
		3- Citation impact factor for the journal in which an article appeared	(-1) (0) (1)	(-1) (0) (1)	(-1) (0) (1)	
		4- Is the raw data from the study available?	(-1) (0) (1)	(-1) (0) (1)	(-1) (0) (1)	
		5- Training of treatment implementers (1. Specified; 2. Not enough data)	(-1) (0) (1)	(-1) (0) (1)	(-1) (0) (1)	
		6- APA format	(-1) (0) (1)	(-1) (0) (1)	(-1) (0) (1)	
		- *Specify another possibility*				
**SUBSTANTIVE CHARACTERISTICS**	**SAMPLE**	7- Did the study report participant age? (Range) reported: Y/N	(-1) (0) (1)	(-1) (0) (1)	(-1) (0) (1)	
		8- Age (mean)	(-1) (0) (1)	(-1) (0) (1)	(-1) (0) (1)	
		9- Age (standard deviation)	(-1) (0) (1)	(-1) (0) (1)	(-1) (0) (1)	
		10- Cultural origin (1. Only one; 2. More than one; 3. Not enough data)	(-1) (0) (1)	(-1) (0) (1)	(-1) (0) (1)	
		11- Socioeconomic level (1. Low; 2. Medium; 3. High).	(-1) (0) (1)	(-1) (0) (1)	(-1) (0) (1)	

		- *Specify another possibility*				
	**SETTING**	12- Implementation context (1. Urban; 2. Rural; 3. Mixed)	(-1) (0) (1)	(-1) (0) (1)	(-1) (0) (1)	
		13- Intervention field (1. Inpatient clinical; 2. Educational; 3. Social; 4. Outpatient clinical; 5. Organizational; 6. Other)	(-1) (0) (1)	(-1) (0) (1)	(-1) (0) (1)	
		14- The authors report the country in which the study was conducted	(-1) (0) (1)	-1) (0) (1)	(-1) (0) (1)	
		- *Specify another possibility*				
	**TREATMENT**	15- Theoretical orientation (1. Specified; 2. Inferred; 3. Not enough data)	(-1) (0) (1)	(-1) (0) (1)	(-1) (0) (1)	
		16- Previous empirical evidence (1. Specified; 2. Not enough data)	(-1) (0) (1)	(-1) (0) (1)	(-1) (0) (1)	
		17- Period of treatment (quantitative time)	(-1) (0) (1)	(-1) (0) (1)	(-1) (0) (1)	
		18- Degree of treatment intensity (i.e. number of dosages)	(-1) (0) (1)	(-1) (0) (1)	(-1) (0) (1)	
		19- Units (1. In group; 2. Individual)	(-1) (0) (1)	(-1) (0) (1)	(-1) (0) (1)	
		20- Strengths and weakness of treatment are discussed (Y/N)	(-1) (0) (1)	(-1) (0) (1)	(-1) (0) (1)	
		- *Specify another possibility*				
**METHODOLOGICAL CHARACTERSTICS**		21- Inclusion and exclusion criteria for units; provided (Y/N)	(-1) (0) (1)	(-1) (0) (1)	(-1) (0) (1)	
		22- Random assignment of units (1. None and without control of extraneous variables; 2. None but with control of extraneous variables; 3. Yes)	(-1) (0) (1)	(-1) (0) (1)	(-1) (0) (1)	
		23- Methodology or design: 1. Experimental; randomized; 2.Quasi-experimental (two groups without randomized assignment) non-equivalent control groups with pre-test and post-test; 3. Pre-experimental (only one group, one measure) / others (questionnaires/observational/naturalistic)	(-1) (0) (1)	(-1) (0) (1)	(-1) (0) (1)	
		24- Sample size (1. $n \leq 15$; 2. $15<n<30$; 3. $n \geq 30$)	(-1) (0) (1)	(-1) (0) (1)	(-1) (0) (1)	
		25- Did the authors say they did a power analysis to calculate sample size? (Y/N)	(-1) (0) (1)	(-1) (0) (1)	(-1) (0) (1)	

	26- Attrition (1. ≤30%; 2. >30%)	(-1) (0) (1)	(-1) (0) (1)	(-1) (0) (1)	
	27- No attrition occurred (Y/N)	(-1) (0) (1)	(-1) (0) (1)	(-1) (0) (1)	
	28- Attrition between groups (1. Homogeneous; 2. Non-homogeneous)	(-1) (0) (1)	(-1) (0) (1)	(-1) (0) (1)	
	29- Exclusions after randomization (Y/N) (specify number)	(-1) (0) (1)	(-1) (0) (1)	(-1) (0) (1)	
	30- How long were units studied before treatment implementation? (1. ≤6 months; 2. 6-12 months; 3. ≥12 months)	(-1) (0) (1)	(-1) (0) (1)	(-1) (0) (1)	
	31- Follow-up period (1. ≤6 months; 2. 6-12 months; 3. ≥12 months)	(-1) (0) (1)	(-1) (0) (1)	(-1) (0) (1)	
	32- Occasions of measurement on each variable (specify number; 1. Post intervention only; 2. Pre and post intervention)	(-1) (0) (1)	(-1) (0) (1)	(-1) (0) (1)	
	33- Measures in pre-test appear in post-test (1. None; 2. Some; 3. All of them)	(-1) (0) (1)	(-1) (0) (1)	(-1) (0) (1)	
	34- Standardized dependent variables: (1. Without: self-reports and post hoc records; 2. Standardized questionnaires or standardized self-reports)	(-1) (0) (1)	(-1) (0) (1)	(-1) (0) (1)	
	35- Intervention context homogeneity (1. Subjects do not receive the treatment in the same contextual conditions; 2. Subjects receive treatment in the same contextual conditions)	(-1) (0) (1)	(-1) (0) (1)	(-1) (0) (1)	
	36- Control techniques: 1. Blinding (beneficiaries); 2. Blinding (implementers); 3. Both; 4. Other (need to specify)	(-1) (0) (1)	(-1) (0) (1)	(-1) (0) (1)	
	37- Construct definition of outcome (1. Replicable by reader in own setting; 2. Vague definition; 3. No definition)	(-1) (0) (1)	(-1) (0) (1)	(-1) (0) (1)	
	38- Statistical methods for imputing missing data (Y/N; Specify)	(-1) (0) (1)	(-1) (0) (1)	(-1) (0) (1)	
	39- Specification of confidence intervals in statistical analysis (Y/N)	(-1) (0) (1)	(-1) (0) (1)	(-1) (0) (1)	
	40- Effect size value	(-1) (0) (1)	(-1) (0) (1)	(-1) (0) (1)	
	41- Effectiveness of treatment (1. Positive effects; 2. Negative effects; 3. Both; 4. None	(-1) (0) (1)	(-1) (0) (1)	(-1) (0) (1)	
	42- Interpretation of results (1. All; 2. Some of them; 3. None)	(-1) (0) (1)	(-1) (0) (1)	(-1) (0) (1)	

	43- Discussion of bias and limitations (1. All; 2. Some of them; 3. None)	(-1) (0) (1)	(-1) (0) (1)	(-1) (0) (1)	
	- *Specify another possibility*				
