# Supplementary material for: The Development of a Checklist to Enhance Methodological Quality in Intervention Programs
Source: Front Psychol. 2016 Nov 18;7:1811. doi: 10.3389/fpsyg.2016.01811 (PMC5114299; doi:10.3389/fpsyg.2016.01811)
Supplement: Supplementary file 5 [file Table_5.PDF]

Supplementary Material

The development of a checklist to enhance methodological quality in intervention programs

Supplementary Table 5. References of pilot studies carried out by the authors in which previous versions of the final proposed checklist have been tested

REFERENCE	ISSUE / DATE SEARCH	ITEMS* (LITERAL)	ITEMS* (SIMILAR)
**1.** Chacón, S., Alarcón, D., Sánchez-Meca, J., & Marín, F. (2002). Evaluative results generalization, design quality and meta-analysis. Main characteristics of intervention designs of published papers in the European context. Paper presented at the *II Annual Campbell Collaboration Colloquium*. Baltimore, MD.	General / until 2002	1, 7, 8, 22, 23, 31, 33, 36, 40	13, 24, 28, 32, 34
**2**. Chacón, S., García, L., Alarcón, D., & Sanduvete, S. (2003, October). Quality of intervention programs for elderly people. A comparative study between the USA and Europe (European Union Countries). Paper presented at the *XI Cochrane Colloquium*. Barcelona, Spain.	Elderly people / until 2002	1, 7, 8, 22, 23, 31, 33, 36, 40	13, 24, 28, 32, 34
**3**. Chacón, S., García, L., Alarcón, D., Sanduvete, S., & Rodríguez, J. A. (2003, November). Systematic reviews and quality design in interventions with elderly people. Poster 2003-X011, presented at the *VII Research Meeting in Nursing*. Zaragoza, Spain.	Elderly people / until 2002	1, 7, 8, 22, 23, 31, 33, 36, 40	13, 24, 28, 32, 34

**4**. Chacón, S., Sánchez-Meca, J., Alarcón, D., Marín, F., Sanduvete, S., & Huedo, T. (2004, February). Quality of program interventions. A comparative study between the USA and Europe (European Union Countries). Paper presented at the *IV Annual Campbell Collaboration Colloquium*. Washington DC.	General / until 2003	22, 23, 26, 31, 40	24, 33, 34
**5**. Sanduvete, S., Chacón, S., & Alarcón, D. (2004, June). Methodological advances to improve quality in intervention programs for elderly people. Paper presented at the *XLVI Congress of Geriatric and Gerontologist Spanish Society*. Las Palmas de Gran Canaria, Spain.	Elderly people / until May, 2004	1, 7, 8, 22-24, 26, 31-34, 36, 40	13
**6**. Chacón, S., Sanduvete, S., & Alarcón, D. (2004, September). Enhancing decision making based on evidence in program evaluation in the European Context. Paper presented at the 6th *Conference of the European Evaluation Society*. Berlin, Germany.	General / until 2004, July	1, 7, 8, 13, 22, 23, 26, 31, 33, 36, 40	24, 32, 34
**7**. Sanduvete, S. (2004). Quality of life in elderly people. *Apuntes de Psicología, 22*(2), 277-288.	Elderly people / until 2003	23, 31-33, 36	
**8**. Chacón, S., Sanduvete, S., & Alarcón, D. (2005, February). Towards the validation of a scale to measure the quality of primary studies for meta-analysis. Paper presented at the *V Annual Campbell Collaboration Colloquium*. Lisbon, Portugal.		22, 23, 26, 31, 40	24, 33, 34

**9**. Sanduvete, S., Holgado, F. P., Chacón, S., & Barbero, M. I. (2005, May). Methodological advances in program designs for elderly people. Paper presented at the *X Spanish Conference of Biometry*. Oviedo, Spain.	Elderly people / until August 2005	1, 7, 8, 22-24, 26, 31-34, 36, 40	13
**10**. Sanduvete, S., & Chacón, S. (2005, June). Action protocol in elderly people intervention design. Paper presented at the *XLVII Congress of Geriatric and Gerontologist Spanish Society*. Malaga, Spain.	Elderly people / until May, 2005	33, 34, 36, 40	
**11**. Chacón, S., Sanduvete, S., & Alarcón, D. (2005, July). Measuring the methodological quality of primary studies in meta-analysis. Paper presented at the *9th European Congress of Psychology*. Granada, Spain.		22, 23, 26, 31, 40	24, 33, 34
**12**. Chacón, S., Sanduvete, S., Holgado, F. P., & Barbero, M. I. (2005, September). Action protocol in a high-quality elderly people intervention design. Paper presented at the IX *Congress of Methodology in Health and Social Sciences*. Granada, Spain.	Elderly people / until August 2005	1, 7, 8, 22-24, 26, 31-34, 36, 40	13
**13**. Shadish, W. R., Chacón-Moscoso, S., & Sánchez-Meca, J. (2005). Evaluation results in Europe evidence-based decision making: enhancing systematic reviews of program. *Evaluation, 11*(1), 95-109.	General / until 2004	1, 8, 23, 31	13, 22, 24, 26, 32-34, 40
**14**. Sanduvete, S., Chacón, S., Alarcón, D., & Sánchez-Meca, J. (2006, February). Methodological advances to enhance quality in intervention programs for elderly people. Poster presented at the *VI Annual Campbell Collaboration Colloquium*. Los Angeles, CA.	Elderly people / until 2005	1, 7, 8, 22-24, 26, 31-34, 36, 40	13

**15**. Sanduvete, S., Chacón, S., Holgado, F. P., Gómez, N., & Sánchez-Martín, M. (2006, July). Causal analysis in training program evaluation. Poster presented at the symposium on *Analysis of causal effects in experimental and quasi-experimental desi*gns. Schloss Dornburg, Germany. Retrieved from http://www.metheval.uni-jena.de/projekte/symposium2008/program.php	Training programs / until May 2006	1, 7, 8, 17-19, 21, 22, 32, 34, 38, 40	13, 23, 24, 26, 28, 29, 31, 33, 36, 37
**16**. Sanduvete, S. (2006). Estudio bibliográfico: características metodológicas de los programas de intervención en la población general y comparación con los dirigidos concretamente a personas mayores [A bibliographic study: Methodological characteristics of intervention programs in general population and comparison with elderly people intervention programs]. In S. Sanduvete, *Methodological advances to improve interventions in elderly people* (73-92). Unpublished research work, University of Seville, Spain.	General and elderly people / until July 2005	1, 8, 13, 22, 23, 26, 31-34, 36, 40	7, 24
**17**. Sanduvete, S., Chacón, S., & Sánchez-Martín, M. (2007, February). Training program design and evaluation: state of the art and improvements. Paper presented at the *X Congress of Methodology in Health and Social Sciences*. Barcelona, Spain.	Training programs / until 2006	7, 40	1, 8, 13, 17-19, 21-24, 26, 28, 29, 31-34, 36-38
**18**. Chacón, S., Sanduvete, S., Sánchez-Martín, M., & Sánchez-Meca, J. (2007, May). Enhancing utility of systematic reviews from methodological quality of primary studies. A case study in training programs. Paper presented at the *VII Annual Campbell Collaboration Colloquium*. London, UK.	Training programs / until 2006	7, 40	1, 8, 13, 17-19, 21-24, 26, 28, 29, 31-34, 36-38

**19**. Sanduvete, S., Chacón, S., Pérez-Gil, J. A., Holgado, F. P., Sánchez-Martín, M., & Lozano, J. A. (2008, July). Methodological improvements in training programs. A scale applied to measure quality. Paper presented at the *III European Congress of Methodology*. Oviedo, Spain.	Training programs / until May 2008	7, 40	1, 8, 13, 17-19, 21-24, 26, 28, 29, 31-34, 36-38
**20**. Sanduvete, S., Chacón, S., Sánchez-Meca, J., Pérez-Gil, J. A., Holgado, F. P., Sánchez-Martín, M., & Lozano, J. A. (2008, July). Effectiveness of training programs: a meta-analysis. Paper presented at the *II Symposium on Analysis of Causal Effects in Experimental and Quasi-experimental Desi*gns. Schloss Dornburg, Germany. Retrieved from http://www.metheval.uni-jena.de/projekte/symposium2008/program	Training programs / until July 2008	23	7, 8, 13, 21, 24, 26, 29, 31, 32, 34, 36, 37, 40
**21**. Sanduvete, S., Chacón, S., Sánchez-Meca, J., Pérez-Gil, J. A., Holgado, F. P., Sánchez-Martín, M., & Lozano, J. A. (2008, July). Effectiveness of training programs: a meta-analysis. Paper presented at the *III European Congress of Methodology*. Oviedo, Spain.	Training programs / until July 2008	23	7, 8, 13, 21, 24, 26, 29, 31, 32, 34, 36, 37, 40
**22**. Sanduvete, S. (2008). Estado de la cuestión en evaluación de la formación continua: aplicación de la escala de calidad [State of the art in training programs evaluation: implementation of a quality scale]. In S. Sanduvete, *Methodological innovations in training program evaluation* (pp. 135-177). Doctoral dissertation. Seville, Spain: University of Seville.	Training programs / until 2007	7, 40	1, 8, 13, 17-19, 21-24, 26, 28, 29, 31-34, 36-38

**23**. Sánchez-Martín, M., Chacón, S., Sanduvete, S., Pérez-Gil, J. A., Lozano, J. A., & Sánchez-Meca, J. L. (2009, September). Methodological indicators to increase quality from the point of view of effectiveness based on meta-analytic studies. Invited paper at the symposium *Applied research and methodological research using designs based on evaluation change*, coordinated by Paula Fernández García. *XI Congress of Methodology in Health and Social Sciences.* Malaga, Spain.		7, 40	1, 8, 13, 17-19, 21-24, 26, 28, 29, 31-34, 36-38
**24**. Sanduvete, S., Chacón, S., Sánchez-Meca, J., Pérez-Gil, J. A., Holgado, F. P., Sánchez-Martín, M., & Lozano, J. A. (2009, September). Effectiveness in training programs: a meta-analysis. Invited paper at the symposium *Innovations in meta-analysis*, coordinated by Julio Sánchez-Meca. *XI Congress of Methodology in Health and Social Sciences.* Malaga, Spain.	Training programs / until Septem-ber 2009	7, 8, 13, 17-19, 22, 23, 32, 38	21, 24, 26, 28, 29, 31, 33, 34, 36, 37, 40
**25.** Sanduvete, S., Chacón, S., Sánchez, M., Pérez, J. A., Holgado, F. P., Lozano, J. A. & Muñoz, N. (2010, July). Measuring methodological quality in primary studies: A content validity study. Paper presented at the *Simpusium on Causality in Educational Research*. Schloss Dornburg, Germany. Retrieved from http://www.metheval.uni-jena.de/projekte/symposium2010/program.php. Copyright: University of Jena, Germany.		1-43	

**26.** Chacón, S., Sanduvete, S. Shadish, W. R., Sánchez, M. y Muñoz, N. (2011, julio). Measuring methodological quality in primary studies: a content validity study revised. Invited paper at the symposium *Methodological innovations in program evaluation. Design and measurement,* coordinated by Salvador Chacón Moscoso and María Teresa Anguera Argilaga. *XII Congress of Methodology in Health and Social Sciences.* Donostia. ISBN: 978-84-694-5058-1.		1-43	
**27.** Sanduvete, S., Chacón, S. & Sánchez, M. (2012, July). Quality of primary studies in meta-analysis: Content validity. Invited paper presented at the Symposium *Methodological innovations in program evaluation*, coordinated by Salvador Chacón Moscoso, M. Teresa Anguera Argilaga & Susana Sanduvete Chaves. *V European Congress of Methodology*. Santiago de Compostela. ISBN: 978-84-9887-921-6.		1-43	
**28.** Sánchez, M., Chacón, S., Sanduvete, S. & Sánchez-Meca, J. (2012, July). Checklist to measure the quality of primary studies in meta-analysis: Inter-coder reliability. Invited paper presented at the Symposium *Methodological innovations in program evaluation*, coordinated by Salvador Chacón Moscoso, M. Teresa Anguera Argilaga & Susana Sanduvete Chaves. *V European Congress of Methodology*. Santiago de Compostela, Spain. ISBN: 978-84-9887-921-6.	Training programs / from 2007 until March, 2011	1, 8, 13, 19, 22-24, 26, 28, 29, 31-34, 36-38, 40	9, 17, 18, 21

**29.** Portell, M., Anguera, M. T., Chacón, S. & Sanduvete, S. (2012, July). Reporting guidelines for evaluations based on low-intervention designs. Invited paper presented at the Symposium *Methodological innovations in program evaluation*, coordinated by Salvador Chacón Moscoso, M. Teresa Anguera Argilaga & Susana Sanduvete Chaves. *V European Congress of Methodology*. Santiago de Compostela, Spain. ISBN: 978-84-9887-921-6.			12, 15-20, 32, 34, 36, 42, 43
**30.** Chacón, S. & Sanduvete, S. (2013, August). Enhancing methodological quality in studies of intervention programs. Invited talk in the opening session of the *Measurement and Statistics program's 2013-14 colloquium series. Florida State University*. Tallahassee, FL.		1-43	
**31.** Sánchez, M., Chacón, S., Sanduvete, S. y Barbero, M. I. (2013, septiembre). Fiabilidad y validez de una escala para medir calidad metodológica en estudios primarios en meta-análisis [Reliability and validity of a scale to measure methodological quality in primary studies in meta-analysis]. Invited paper presented at the symposium Advances in meta-analysis, coordinated by Julio Sánchez-Meca. *XIII Congress of Methodology in Health and Social Sciences*. San Cristóbal de La Laguna, Spain.	Training programs / from 2007 until March, 2011	1, 8, 13, 17-19, 21-24, 26, 28, 29, 31-34, 36-38, 40	9
**32.** Portell, M., Chacón, S., Sanduvete, S. y Anguera, M. T. (2013, septiembre). Towards a guideline for conducting and reporting observational studies in the field of program evaluation. Invited paper presented at the symposium *Methodological innovations in program evaluation*, coordinated by Salvador Chacón Moscoso y Susana Sanduvete Chaves. *XIII Congress of Methodology in Health and Social Sciences.* San Cristóbal de La Laguna, Spain.			12, 15-20, 32, 34, 36, 42, 43

**33.** Chacón, S., Sanduvete, S., Portell, M. & Anguera, M. T. (2013). Reporting a program evaluation: Needs, program plan, intervention, and decisions. *International Journal of Clinical and Health Psychology, 13*(1), 58-66**.**			7-11, 15, 16, 34, 35, 41
**34.** Sanduvete, S., Chacón, S. & Becker, B. J. (2014, July). Randomized vs. non-randomized studies to assess training programs effectiveness: a meta-analysis. Invited poster presented at the *IX Annual Meeting of the Society for Research Synthesis Methodology*. York, UK.	Training programs / from 2007 to 2012	7, 8, 22, 23, 31, 32, 35	12, 13, 21, 24, 26, 27, 29, 34, 37
**35.** Padilla-Muñoz, E. M., Pozo-Granados, L. Y., Chacón-Moscoso, S., & Sanduvete-Chaves, S. (2015, July). Enhancing methodological quality of evaluations in cognitive-behavioral programs for school children with Attention Deficit Hyperactivity Disorder (ADHD). A case study. Invited paper at the symposium *Innovaciones metodológicas en evaluación de programas: calidad metodológica y eficacia desde la evidencia*, coordinated by Salvador Chacón-Moscoso & Susana Sanduvete-Chaves. *XIV Congreso de Metodología de las Ciencias Sociales y de la Salud*. Palma de Mallorca, Spain. ISBN: PM 744-2015	Attention deficit hyper-activity disorder / from 2011 to 2012	21	23, 32, 41, 42
**36.** Portell, M., Anguera, M. T., Chacón, S., & Sanduvete, S. (2015). Guidelines for reporting evaluations based on observational methodology. *Psicothema, 27*(3), 283-289. doi:10.7334/psicothema2014.276		24	7-12, 17-19, 21, 23, 31, 34, 41
**37.** Sánchez, M., Chacón, S. & Sanduvete, S. (2015). Enhancing quality in training programs: an application in the sport area. *Revista Internacional de Medicina y Ciencias de la Actividad Física y el Deporte*, *15*(60), 613-629.	Training programs in sports / from 2005 to 2006		7-11, 19, 23, 32, 34, 35, 41-43

***** Numbers of items are based on the questionnaire used in the content validity study (Supplementary Table 4).
