# Supplementary material for: The Development of a Checklist to Enhance Methodological Quality in Intervention Programs
Source: Front Psychol. 2016 Nov 18;7:1811. doi: 10.3389/fpsyg.2016.01811 (PMC5114299; doi:10.3389/fpsyg.2016.01811)
Supplement: Supplementary file 7 [file Data_Sheet_2.docx]

**Appendix.** Definitions of the 12 items and their coding criteria for the final proposed checklist

1. **Inclusion and exclusion criteria for the units provided**: The reasons why some people were able to participate in the study and others were not were stated explicitly:
   1. **Yes:** Adequate selection criteria for units and applied to all the potential participants.
   2. **No:** Inadequate selection criteria for units and/or with exceptions in their application.
2. **Methodology or design:** Something an experimenter could manipulate or control in an experiment to help address a threat to validity (Shadish, Cook, & Campbell, 2002, p. 507):
   1. **Experimental; randomized:** An experiment (exploration of the effects of manipulating a variable; Shadish et al., 2002, p. 507) in which units are randomly assigned to conditions (Shadish et al., 2002, p. 511).
   2. **Quasi-experimental (two groups without randomized assignment) non-equivalent control groups with pre-test and post-test:** An experiment (exploration of the effects of manipulating a variable; Shadish et al., 2002, p. 507) in which units are not randomly assigned to conditions (Shadish et al., 2002, p. 511).
   3. **Pre-experimental/others (questionnaires/observational/naturalistic)** (Olivares, Rosa, & Sánchez-Meca, 2000; Sánchez-Meca, 1997): A study in which there is only one group and one measure; or when there was no intervention, but recordings were made of the participants’ behavior, whether this was spontaneous or elicited (usually by means of questions).
3. **Attrition:** Loss of units. In randomized experiments, this refers to loss that occurred after the random assignment had taken place (Shadish et al., 2002, p. 505); percentage of the initial sample that did not conclude the study.
4. **Attrition between groups:** This item evaluated the differences in attrition between two groups (given in percentage).
5. **Exclusions after randomization (specify percentage):** This is also called post-assignment attrition and includes cases in which, after assigning a participant to a condition, an experimenter deliberately dropped that participant from the data (Shadish et al., 2002, p. 323).
6. **Follow-up period**: This determined how long measurements were taken after finishing the intervention. When the study presented more than one follow-up period, the longest was recorded (given in number of days).
7. **Occasions of measurement for each variable (specify number)** (Olivares et al., 2000): This item specified when the measurements were taken.
   1. **Pre- and post-intervention:** Some measurements were taken before the intervention and others after it. The specific number of measurements on each occasion was recorded.
   2. **Post-intervention only:** All the measurements were taken after the intervention. The specific number of measurements was recorded.
8. **Measures in pre-test appear in post-test** (Olivares et al., 2000): This item counted the number of measurements that were taken before the intervention and for the remaining measurement occasions.
   1. **All of them (**Olivares et al., 2000): All measurements were taken at all the measurement occasions.
   2. **Some:** At least one of the measurements was taken at all measurement occasions.
   3. **None:** None of the measurements was taken at all measurement occasions.
9. **Standardized dependent variables:** Level of normalization of the tool to measure the variable that varied in response to the independent variable (also called effect or outcome) (Shadish et al., 2002, p. 507).
   1. **Standardized questionnaires or standardized self-reports** (Olivares et al., 2000): At least one measurement was taken using structured tools. The data were gathered using a homogeneous procedure. Some study of their psychometric properties was carried out.
   2. **Without (self-reports and post hoc records) (**Olivares et al., 2000): All the measurements were taken using ad hoc tools, developed in a specific situation, and whose validity was not checked.
10. **Control techniques** (Olivares et al., 2000):
    1. **Double-masking:** Also called double-blind, this refers to a procedure that prevented participants and experimenters from knowing the hypotheses (Shadish et al., 2002, p. 78).
    2. **Masking (beneficiaries):** Also called blind, this refers to a procedure that prevented participants from knowing the hypotheses (Shadish et al., 2002, p. 78).
    3. **Masking (implementers):** Also called blind, this refers to a procedure that prevented experimenters from knowing the hypotheses (Shadish et al., 2002, p. 78).
    4. **Other (need to specify):** In this category, the control techniques were specified when they were not those mentioned in the previous categories (i.e., matching, blocking, or stratifying).
11. **Construct definition of outcome**: Explanation of the concept, model, or schematic idea measured as a dependent variable (Shadish et al., 2002, p. 506):
    1. **Replicable by reader in own setting:** All the concepts measured as dependent variables were defined in a conceptual and empirical way.
    2. **Vague definition:** At least one concept measured as a dependent variable was defined in a conceptual and/or empirical way.
    3. **No definition:** No concept measured as a dependent variable was measured in a conceptual or empirical way.
12. **Statistical methods for imputing missing data**: To estimate what the study would have yielded if there had been no attrition (Shadish et al., 2002, p. 337):
    1. **Yes (specify):** Values for the missing data points were imputed so that they could be included in the analyses. The specific method used was specified, that is, sample mean substitution, last value forward method for longitudinal data sets, hot deck imputation, single imputation, or multiple imputation.
    2. **No:** Attempts were made to estimate effects without imputing missing data.
